# Temperature‐related geographical shifts among passerines: contrasting processes along poleward and equatorward range margins

**DOI:** 10.1002/ece3.1683

**Published:** 2015-10-20

**Authors:** Laura E. Coristine, Jeremy T. Kerr

**Affiliations:** ^1^ Canadian Facility for Ecoinformatics Research Department of Biology University of Ottawa 30 Marie Curie Ottawa Ontario Canada K1N 6N5

**Keywords:** Breeding birds, climate change, climate debt, peripheral populations, range shift, thermal limit

## Abstract

Climate change is causing widespread geographical range shifts, which likely reflects different processes at leading and trailing range margins. Progressive warming is thought to relax thermal barriers at poleward range margins, enabling colonization of novel areas, but imposes increasingly unsuitable thermal conditions at equatorward margins, leading to range losses from those areas. Few tests of this process during recent climate change have been possible, but understanding determinants of species’ range limits will improve predictions of their geographical responses to climate change and variation in extinction risk. Here, we examine the relationship between poleward and equatorward range margin dynamics with respect to temperature‐related geographical limits observed for 34 breeding passerine species in North America between 1984–1988 and 2002–2006. We find that species’ equatorward range margins were closer to their upper realized thermal niche limits and proximity to those limits predicts equatorward population extinction risk through time. Conversely, the difference between breeding bird species’ poleward range margin temperatures and the coolest temperatures they tolerate elsewhere in their ranges was substantial and remained consistent through time: range expansion at species’ poleward range margins is unlikely to directly reflect lowered thermal barriers to colonization. The process of range expansion may reflect more complex factors operating across broader areas of species’ ranges. The latitudinal extent of breeding bird ranges is decreasing through time. Disparate responses observed at poleward *versus* equatorward margins arise due to differences in range margin placement within the realized thermal niche and suggest that climate‐induced geographical shift at equatorward range limits more strongly reflect abiotic conditions than at their poleward range limits. This further suggests that observed geographic responses to date may fail to demonstrate the true cost of climate change on the poleward portion of species’ distributions. Poleward range margins for North American breeding passerines are not presently in equilibrium with realized thermal limits.

## Introduction

Recent climate changes are associated with species’ geographical ranges shifting poleward or upward along elevational gradients (Parmesan 1996; Thomas and Lennon 1999; Hill et al. 2002; Parmesan and Yohe 2003; Brommer 2004; Walther et al. 2005; Zuckerberg et al. 2009; Chen et al. 2011a). Because species’ distributions depend strongly on thermal tolerances (Kukal et al. 1991), these shifts have long been anticipated (Peters and Darling 1985). Yet, climate change rates are spatially heterogeneous (Loarie et al. 2009), which could cause spatial variation in population responses among species. Nevertheless, the pace of anthropogenic climate change may outstrip the capacity of many species to track shifting zones within which climates are tolerable (Bedford et al. 2012; Devictor et al. 2012), leading to expectations that climate change will accelerate extinction rates (La Sorte and Jetz 2010; Maclean and Wilson 2011).

**Figure 1:**
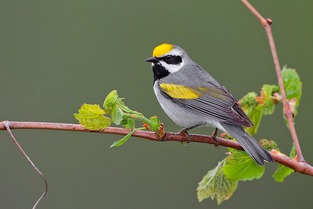
Photograph of a golden‐winged warbler (*Vermivora chrysoptera)* in Pocahontas County, West Virginia, United States. One of the 34 species used in the study. Photo credit: Jacob Spendelow.

Range responses, whether at poleward or equatorward range margins, are thought to depend on the proximity of fundamental thermal niche boundaries (Mayhew et al. 2012; Sax et al. 2013). Much of the current research on thermal limitations at range margins of birds has focused on wintering distributions (La Sorte and Thompson 2007; Zuckerberg et al. 2011; La Sorte and Jetz 2012), where thermal release from cold limitations (viz. minimum winter temperature) promotes poleward range expansion by lowering metabolic requirements (Root 1988). Breeding bird distributions are thought to similarly reflect thermal limitations, and species such as the golden‐winged warbler (Fig. 1; *Vermivora chrysoptera*) have undergone substantial range retraction at equatorward margins. However, direct tests of thermal limits at breeding range margins have been infrequent (but see Melles et al. 2011) despite the potential fitness implications arising from loss or misalignment with thermal niche (Jiguet et al. 2010). Extinction risk increases if species’ climatic niches shift (in geographic space) but species’ populations cannot, leading to compression of their geographical ranges. Climate‐driven extinction is expected to operate among populations where warming causes local thermal conditions to exceed tolerable limits, which is anticipated along equatorward range margins. Range expansion, conversely, is expected along poleward range margins as a function of facultative colonization into new areas where warming has relaxed barriers to dispersal and establishment of new populations (Sunday et al. 2012).

**Figure 2:**
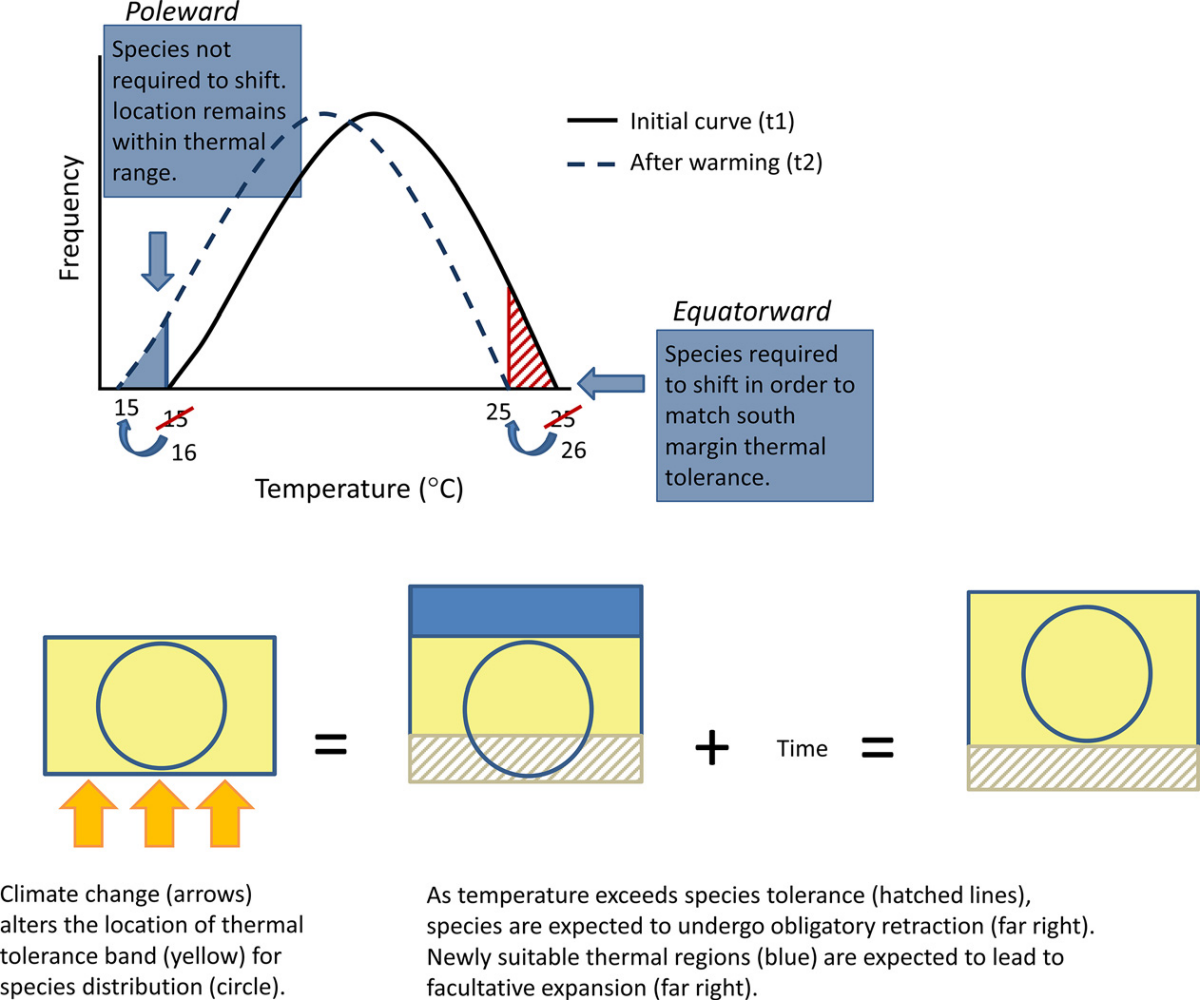
Conceptual diagram of how climate change may limit species’ geographical distribution based on direct or indirect effects of temperatures observed during the breeding season. Poleward range margins are expected to undergo facultative expansion. Equatorward range margins are expected to undergo obligatory retraction.

In other words, different processes likely determine how species’ populations respond to climate change, depending on whether populations are located at poleward or equatorward range margins (Fig. 2). Constraints near species’ range limits can be direct if temperatures exceed thresholds that species tolerate after accounting for behavioral thermoregulation (McKechnie and Lovegrove 2001; Robinson et al. 2007). Further, species’ geographic distribution, which represents the occupied or realized niche, may be placed relative to the fundamental niche so that some portions of the distribution are closer to fundamental niche limits (Araújo et al. 2013; Sax et al. 2013). Poleward range expansion is now widely observed among species in many taxa and predominantly in the direction expected given climate change (Hickling et al. 2005; Maclean et al. 2008; Melles et al. 2011; Sunday et al. 2012). If poleward range limits reflect climate‐related barriers, failure to track warming along these range limits incurs a climate debt (sensu Devictor et al. 2012). Population extinctions due to climate change have been observed in terrestrial ecosystems but are much less well characterized than range expansion along poleward limits (Parmesan and Yohe 2003; Wilson et al. 2005; Maclean et al. 2008; Sunday et al. 2012). Perhaps the most obvious, and an especially longstanding, explanation for such observations is that poleward range limits directly reflect species’ environmental tolerances, particularly to temperature while equatorward range margins reflect biotic interactions (MacArthur 1972; Cahill et al. 2014).

There are several reasons why species may fail to track geographical shifts in their measured thermal niches, including habitat requirements relative to habitat availability (White and Kerr 2007), phenotypic plasticity (Pichancourt and van Klinken 2012), niche dynamics (or the changes in climatic niche relative to geographic placement for a species; La Sorte and Jetz 2012; Monahan and Tingley 2012), dispersal capacity (Bedford et al. 2012), compensatory dynamics (Doak and Morris 2010), and life‐history characteristics (Tingley et al. 2012). Land‐use changes and habitat loss may prevent some species from dispersing rapidly enough to track geographical movement of their climatic (or tolerance, sensu Sax et al. 2013) niches, leading to climate‐induced biotic homogenization as generalists disperse successfully to new locations while specialists do not (White and Kerr 2007). Changes in climatic conditions, including interacting effects of multiple climatic variables (VanDerWal et al. 2013), may also be accompanied by increased frequency and/or intensity of extreme weather events. Extreme events may exceed species’ tolerances and cause population extinction and inhibit or reverse climate‐related range responses (i.e., cause extinction in a population that successfully established beyond the species’ historical range boundary). Adult birds can tolerate temperatures in their breeding range, which may often be cooler than in their overwintering grounds (e.g., Boucher‐Lalonde et al. 2014). However, thermoregulatory constraints during the breeding season (Stein et al. 2010) means that species should have lower tolerance to temperatures that approach thermal niche limits (due to either gradual changes in climate or stochastic changes that arise from weather extremes) during the breeding season (Jankowski et al. 2013). This suggests that for adult birds, thermal limits may vary seasonally (Monahan 2009) due to physiological shifts based on breeding status (Vehrencamp 1982), while juveniles are highly susceptible to temperature‐related mortality due to lower ability to thermoregulate. Juvenile survival rates decrease sharply when temperatures are elevated as during extreme heat events, leading to bird population declines (Albright et al. 2011) regardless of plasticity in adult behaviors in response to warming during nesting (Vedder 2012). The extent to which species’ distributions shift in geographical and niche space, regardless of such behavioral and phenotypic plasticity, will inform potential management interventions and possibly species’ conservation prospects.

Here, we examine range margin dynamics over the complete breeding ranges of a group of intensively sampled bird species in North America relative to substantial climate changes. First, we test whether geographical range limits correspond with geographically defined thermal niche limits (sensu Jiguet et al. 2006) at both the poleward and equatorward limits of breeding ranges and whether the temperature difference between range margins and species’ observed realized thermal niche limits change over time. While climate change may cause the boundaries of species’ realized niches to shift geographically, this effect is not expected to cause range losses from the warmest areas occupied by the species unless conditions in those areas exceed either adult or juvenile thermal tolerances. Breeding season thermal tolerance tends to be narrower than nonbreeding season thermal tolerance due to adult constraints on behavioral thermoregulation during the nesting period (Walsberg and King 1978), juvenile susceptibility to temperature extremes (Albright et al. 2011), and energetic costs associated with reproduction (Tinbergen and Dietz 1994; Golet et al. 2000). If geographic range shifts are strongly driven by temperature and realized niche limits are situated close to fundamental limits, then we expect that as range margin temperatures approach those of the realized thermal niche limit, the probability of species’ range shift increases. In this sense, we investigate, not the acute extremes associated with extreme weather events and to which species may respond through either short‐term adaptive responses or population dynamics that are neither tested nor investigated herein but rather, the thermal niche limits associated with long‐term observations of distribution.

## Methods

We used historic baseline data from 1984 to 1988 to determine species’ realized thermal niche limits. We compared those baseline observations against those from a second, later time period (2002–2006) to assess whether (1) thermal niche limits shifted, (2) range margin temperatures represented thermal niche limits, and (3) range margins had shifted in the direction expected given local climate warming as a function of range margin thermal proximity to the historic realized thermal tolerance limits. All data are published on DRYAD Digital Repository.

### Time period selection

Time periods were selected to maximize potential detectability of climate‐driven range shifts. The available time span was constrained by both climate and BBS (Breeding Bird Survey) limitations. Warming was slight prior to the mid‐1980s and accelerated after this time (IPCC 2013). Sampling among BBS routes was also much less consistent prior to the 1980s (Kendall et al. 1996). To detect climate change‐mediated shifts in range margins (an aggregate of 10 BBS route locations), we required spatially dense data points that could be matched between two time periods.

Samples were aggregated into five‐year time periods, where the range of time within a period was less than the range of time between periods. This increased the number of BBS routes available for matching between time periods and minimized both the effects of natural (i.e., not attributable to climate change) fluctuations in range boundaries (Brown et al. 1996), as well as differences in detectability that may arise due to species, habitat, and observer effects. The greatest number of consistently sampled routes occurred during the time periods of 1984–1988 and 2002–2006.

### Species and study region

Data for passerine breeding distributions were drawn from the North American Breeding Bird Survey (BBS; USGS 2009) in two study periods, 1984–1988 and 2002–2006. The BBS is a standardized annual survey conducted by ornithologists with the skill to identify all birds within a region by both sight and sound. Each route is 39.5 km in length and consists of 50 stops at 0.8‐km intervals. Stops are a three‐minute count of all birds observed or heard within a 0.4‐km radius (USGS 2009). A total of 2018 routes were sampled a minimum of once in each time period and were included here. Breeding birds are subject to heterogeneous detection probabilities that vary among species, habitats, and observers. Each study period extended over 5 years to maximize the likelihood that breeding bird species would be successfully detected, and range margin as well as thermal niche limit estimates was based on an average of 10 occurrence locations, thereby minimizing the effect of nondetection at any single site. Rare and cryptic species, which have lower detection probabilities, were not used in the study.

We did not remove routes sampled by first‐year observers, as this is a correction applied to improve population or abundance trend estimates. BBS observers tend to count fewer individuals of a species during the first year of survey for a route, and this has a minimal (1.8%/year) impact on trend estimates with a greater effect for routes sampled prior to 1970 (Kendall et al. 1996). Fewer than 10% of routes are surveyed by first‐year observers in any given year, and the majority of species, including the majority of species in our study, do not have a demonstrable first‐year observer effect (Sauer et al. 1994; Kendall et al. 1996). Thus, removal of first‐year observer routes would have had a disproportionate effect on number of routes available for this study. Similarly, observer effects associated with age‐related hearing loss also lower count estimates for certain species and may additionally contribute to nondetection (Farmer et al. 2014). First‐year observer and observer effects are not expected to exert a directional bias in range margin location through time, although they could potentially have a minor effect on abundance‐weighted estimates for the few species in our study that are affected. As such, excluding these routes would have weakened potential range shift signal by removing valid occurrences (and nonbiased count estimates) for the majority of species in our study, although this would increase the precision of abundance weighting for the remaining.

**Figure 3:**
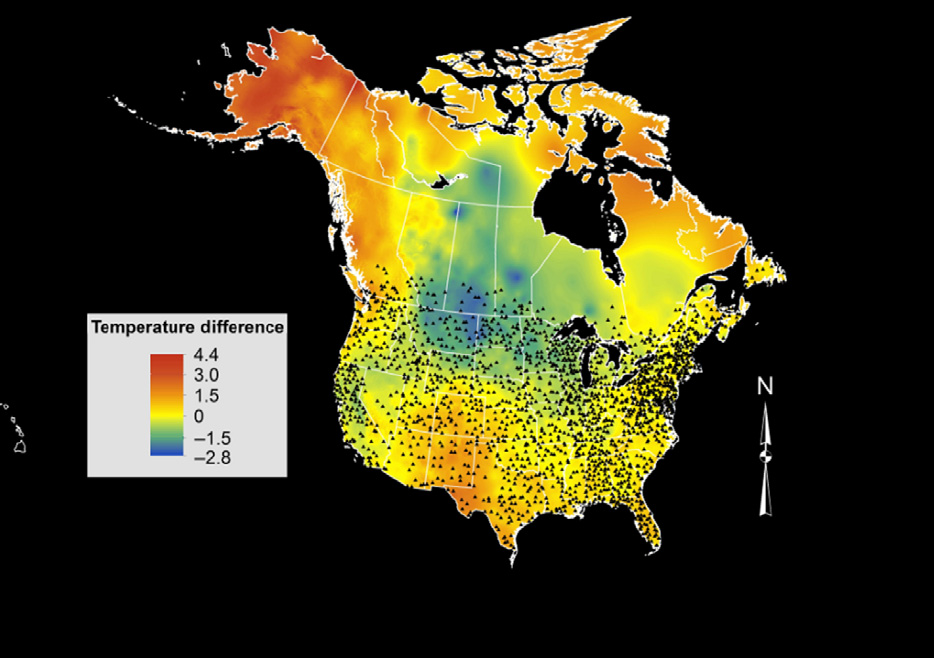
Breeding season temperature difference (°C) across North America between 1984–1988 and 2002–2006, calculated as the means of observed temperatures during April, May, and June of each year within the separate time periods. Overlaid are the locations of breeding bird survey routes that were included in this study for purposes of measuring temperature differences within the ranges of each of the 34 passerine species for which northern and southern breeding range limits were included. Routes located above 52° N latitude were excluded due to low sampling density. Map is projected in Albers Equal Area to improve visual representation of the study region.

The study region was chosen to include only the most densely sampled regions of North America and consists of southern portions of Canada (<52° N latitude) and the contiguous United States (Fig. 3). Despite inconsistent sampling on routes through time, there was no tendency for sampling on poleward routes to differ from those elsewhere from 1984–1988 to 2002–2006 (*t* = −0.036, *P* = 0.97).

Passerines are more readily detected on BBS routes than other avian species (Link and Sauer 1997), and thus, form the focus for this research. Only species whose entire breeding ranges were within the study region were included. Breeding distributions were verified, and bird taxonomy was updated using the Cornell Lab of Ornithology Birds of North America online database The Birds of North America Online (2013). The maximum historical poleward margin breeding limit for species’ inclusion was 49° N latitude (a minimum of 3 degrees latitude, or ~330 km, below the northern study extent) to allow for sampling of routes that could provide available climate space and therefore permit detection of poleward expansion. Equatorward breeding margins were wholly within the relatively densely sampled region of the southern United States.

To control for factors that might interfere with detection of climate‐mediated range shift, stringent species’ selection criteria were applied. Passerine species that were not native to North America were omitted. Species with small population sizes (<100 individuals/time period), highly disjunct populations, species inhabiting principally coastal areas, or rare species (present on fewer than 30 unique routes per period) were omitted to reduce sampling artifacts or the potentially confounding effects of habitat limitations on geographical range boundaries. This left a total of 34 species with breeding ranges entirely contained within the study region and with historical poleward breeding ranges below 49° N latitude (Appendix S1). These species included neotropical migrants (*n* = 26), residents (*n* = 6), and short distance migrants (*n* = 2). The majority of study species had portions of their equatorward margins near the Gulf of Mexico (*n* = 21), but we also examined this group of species separately to evaluate whether this potential geographical artifact altered our conclusions.

### Climate data

Temperature measurements for all BBS routes in each year included in the study were derived from weather station data that were gridded using thin plate spline smoothing algorithms and resampled to a 5 arc‐minute resolution based on cross‐validated determination of climate values' dependence on elevation (McKenney et al. 2011, see Hutchinson 2004 and Xu and Hutchinson 2013 for further details).

Breeding season temperature measurements were based on average April, May, and June temperatures (i.e., the breeding season for these birds) observed within each time period using values from years in which each species was observed. We accounted for spatial differences in the start of breeding seasons among them by measuring temperature averages of April, May, and June temperatures, which coincide with peak breeding seasons in the North American ranges of these species, based on observed inclusion in the BBS. Although some birds breed in July and August, we excluded these months on the basis that site selection for nesting is not informed by later season temperatures, except insofar as temporal autocorrelation exists within temperature measures for a given location. Route centroids were calculated for each route using ArcGIS (ESRI 2010), and each route was then associated with the mean breeding season temperature calculated within a 20‐km buffer around the centroid, thus including the entirety of every 39.5 km route. Significant temperature changes were observed between the two periods selected for this study (+0.43°C across North America over the 18 year study period; see Fig. 3).

### Range margin and thermal niche data

Temperatures along species’ breeding range margins were compared against the highest and lowest April–June temperatures where breeding was observed for each species. This observation period includes the peak breeding season for each species included here, although those seasons extend over longer time periods (ranging between March to September) (The Birds of North America Online, 2013). Our hypothesis is that the hottest and coolest temperatures associated with nesting represent the extremes that these species can tolerate in practice, given the array of biotic interactions experienced in the field (i.e., their realized thermal niche limits, at least for juveniles). If this hypothesis is incorrect, warming will not be associated with shifts in species’ geographical or thermal limits through time. Conversely, the hypothesis would be supported if species’ ranges track shifting climatic conditions temporally. Temperatures observed along species’ equatorward range margins (i.e., from the most southerly BBS routes where the species was observed) were compared with the warmest breeding season temperatures observed at routes where the species was confirmed to be present within its range (their hypothesized warm realized thermal limit). Conversely, poleward range margin temperatures were compared against minimum temperatures observed at any BBS site where the species was confirmed to be present (their hypothesized cool realized thermal limit). The warm and cool realized thermal limits were selected from the pool of historic breeding season temperatures where a species was observed, and represent the mean breeding season temperature for the 10 warmest or coolest locations. The warm and cool thermal values are more representative of distributional thermal extremes. There is some literature to suggest that passerine distributions are resilient to temperature extremes that are of short duration or intensity (Pipoly et al. 2013; Malinowska et al. 2014; Villen‐Perez and Carrascal 2014). Given that our study incorporates 5 years of data to determine thermal limits in breeding distribution, our determination of thermal limit temperatures are more representative of seasonal temperature exposure over longer time periods and how these relate to distributional limits. Temperature extremes impact species’ distributions through acute exposure to intolerable temperature over very short time periods rather than to the more gradual trend of warming that is normally examined in studies that investigate climate change impacts on biodiversity distributions.

This analysis tests (1) how closely temperatures at breeding range margins relate to the coolest and warmest of average breeding season temperatures (hereinafter referred to as temperature limit or thermal niche limit) anywhere within the species’ breeding range, (2) whether these range margin temperatures could approximate the temperature limits these species can tolerate in the field, and consequently (3) whether range shifts could arise because warming forces range loss (at the equatorward margin) or facilitates colonization of previously unoccupied areas (at the poleward margin). We expected that BBS routes with the most extreme temperatures would be found along the range margins themselves. Species’ presence was recorded based on occurrence on a route within the five‐year time period, and abundance‐weighted mean breeding season temperature was calculated based on years within those time periods that each species was actually observed. We calculated abundance weighting by multiplying the average breeding season temperature on the route for years where the species was observed by the abundance in the years of observation, divided by the total abundance for the species’ range margin or thermal limit. Thus, routes with a higher recorded abundance of individuals were weighted more heavily.

Range margins were defined as the 10 most poleward or equatorward routes where a species was present within each time period. Change in the geographical position of species’ range margins were calculated as poleward displacement (in kilometers) of the respective range margin for each passerine species based on the difference between the two‐five‐year time periods, 1984–1988 and 2002–2006. The boundaries of realized thermal niches were calculated for each species during both time periods based on the average of 10 coolest or warmest routes from which the species was recorded (see also Jiguet et al. 2006). These measurements of niche limitations may correspond to species’ realized thermal niche limits but more specifically reflect species’ tolerance niches (Sax et al. 2013). Temperatures along range margins were calculated using the 10 most poleward or equatorward BBS routes where a given species was observed in either time period. Temperatures for these routes were only measured in years when the species was observed.

### Statistical analyses

**Figure 4:**
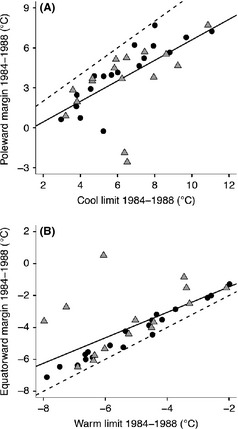
Relationship between mean breeding season temperature at the (A) cool edge and (B) warm edge of the realized niches (calculated as the 10 coldest/warmest routes with species’ occurrence during 1984–1988) and mean breeding season temperature at the range margin expected to correspond to the niche edge (calculated as the 10 poleward or equatorward routes with species’ occurrence during 1984–1988). The black line represents the observed relationship, and the dashed line represents the expected relationship if there is perfect correspondence between thermal niche limits and species’ range margin. Circular data points represent species that are shifting as expected based on temperature change. Temperature was corrected for total niche breadth.

**Figure 5:**
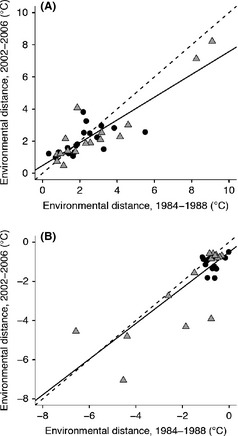
Temporal relationship in environmental distance calculated as the difference in mean breeding season temperature (°C) separating the thermal niche boundary and range margin in 1984–1988 and 2002–2006 for (A) the poleward margin and cool niche boundary, and (B) the equatorward margin and warm niche boundary. Points above the 1:1 line in (A) represent species for which the temperature difference between their cold niche limits and poleward range margin became larger through time. Points below the 1:1 line in (B), conversely, represent species that had populations closer to their warm niche limits in the early time period than in the later one. The black line represents the observed relationship, and the dashed line represents the expected relationship. Triangular data points indicate species whose margin shifted equatorward, while circular data points indicate species whose margin shifted poleward.

Geographical data were processed using ArcGIS 10.0 (ESRI 2010), and all statistical analyses were performed using R, version 3.01 (R Core Team 2013). For each species in the 1984–1988 time period, we examined the relation between mean breeding season temperature at the 10 coolest routes and mean breeding season temperature at the 10 most poleward routes relative to species’ thermal breadth (i.e., the temperature deviation at the coldest or geographically most extreme locales from the thermal niche centroid). By adjusting for the gap between thermal niche centroid and the temperatures along range margins or along niche boundaries, we reduce variability that may arise because of differences in the breadth of each species’ thermal niches. For example, we anticipate that species with narrow thermal niches might be more susceptible to small climate changes than species with very broad thermal niches. Differences between temperatures at range margins (equatorward or poleward) and most extreme breeding season temperatures (either warmest or coldest) at sites occupied by the 34 breeding bird species were examined using t‐tests. This analysis tests whether species’ range limits coincide with realized temperature limits that could govern geographical range responses. We repeated this analysis to test the relationship between equatorward and warm niche limits in the historical time period. Thermal niche centroid was calculated as the abundance‐weighted average breeding season temperature observed for a species (Maclean et al. 2008). We also tested for correspondence between species’ realized thermal niche limits (as inferred based on observed breeding presences) and the temperatures at species’ poleward and equatorward margins (defined here as environmental distance) using ordinary least squares regression (see Fig. 4). Finally, having determined the temperature gap between range margins and the temperature limits within each species’ breeding range, we tested whether the magnitude of this temperature gap changed through time, also using regression (see Fig. 5).

Using logistic regression, we tested whether the geographical range responses (i.e., colonization of historically unoccupied areas at the poleward range margin, or a population extinction at the equatorward range margin) to climate change was more likely when breeding season temperatures at range margins approached the bounds of the realized thermal niche. Geographic range shift was set as a binary variable and was coded as 1 when geographical range shifts matched the expected direction based on change in temperature and 0 when they did not (see Fig. 7). This allowed us to account for local climate differences on range margins regardless of whether temperatures warmed (which we expected would cause population losses at sites along equatorward range margins or colonization of new areas at the poleward edge) or cooled (which would create the opposite expectation).

The BBS is not readily capable of measuring population trends within or between species when observer effects and heterogeneous detection probabilities are not accounted for (Sauer et al. 1994; Kendall et al. 1996; Sauer et al. 2004). However, we did explore whether substantial abundance changes resulted from climate change from 1984–1988 to 2002–2006 at species’ poleward and equatorward range limits and at warm and cool realized thermal niche limits, respectively, holding historical locations constant (Supplemental Materials). Species absence in the second time period indicates local population extinction, and thus, we examined change in abundance to account for local extinction of populations by averaging the temperature change and abundance change with these values included. We re‐examined these relationships after excluding localities where population extinctions were observed because of the possibility that apparent population extinction could be confounded with failure to detect the species on the BBS route.

## Results

Breeding season temperatures at poleward margins were not the coldest sites occupied by a species in 1984–1988 (two sided *t* = 7.61, df = 33, *P*‐value <10^−6^). However, there was a strong, positive, linear relationship between temperatures observed along poleward range margins and routes where the coldest temperatures were observed (Fig. 4A, *R* ^2^ = 0.44, *P*‐value <10^−6^). Poleward breeding range margins do not appear to be directly limited by cool breeding season temperatures. Mean breeding season temperatures in 1984–1988 were somewhat cooler than expected at equatorward margins (Fig. 4B; *R* ^2^ = 0.50, *P*‐value <10^−6^) if that part of the species’ range is directly limited by maximal thermal niche limits (*t*‐test: two sided *t* = −5.03, df =  33, *P*‐value <10^−6^), despite a strong relationship between these values. A high proportion of species have a narrow environmental distance between warm thermal niche limits and equatorward range margin temperatures. These results were not affected if only neotropical migrants or species partially bounded by the Gulf of Mexico were considered separately (see Fig. S1a–d; Appendix S1). Species that were geographically constrained by the Gulf of Mexico at their equatorward range margin had a stronger correlation between equatorward margin temperature and the warmest temperatures tolerated within their range.

There was a strong temporal relationship in temperature difference (in °C for mean breeding season temperature) between the poleward margin and cool thermal niche edge from 1984–88 to 2002–2006 (Fig. 5A; *R* ^2^ = 0.74, *P*‐value <10^−6^), and this remained consistent through time (*t*‐test: two sided *t* = 1.52, df = 33, *P*‐value =0.14). That is, the realized thermal niche limits were not significantly “closer” (measured as environmental distance) to mean breeding season temperatures at the poleward range margin in 2002–2006 than they were in 1984–1988. If species were failing to track warming temperatures, poleward margin temperatures should have been further from realized thermal limits in the later time period. Similarly, the temperature difference between equatorward margin and warm thermal niche edge in 1984–1988 was strongly related to the difference in 2002–2006 (Fig. 5B; *R* ^2^ = 0.66, *P* < 10^−6^). Along the equatorward range margins, species were slightly further from their thermal niche limits in 2002–2006 than they were in 1984–1988 (*t* = 2.64, two tailed, df = 33, *P*‐value = 0.013). Neotropical migrants showed similar correspondence at equatorward and warm thermal niche limits, and at poleward and cool thermal limits, but the environmental distance separation was not significantly different through time. Species with equatorward range margins partially along the Gulf of Mexico were closer to their measured upper thermal niche margins than other species in both time periods, and this difference did not change through time (see Fig. S2a‐d; Appendix S1).

**Figure 6:**
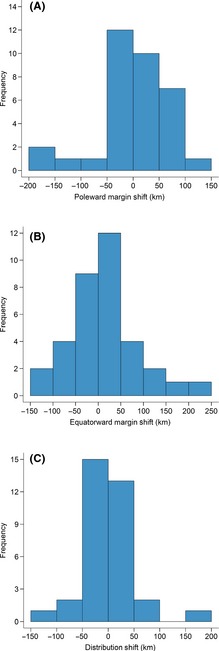
Frequency distribution of rate of latitudinal range margin shift at (A) the pole‐ward margin and (B) the equatorward margin from 1984–1988 to 2002–2006. Positive values represent pole‐ward shift at the range margin, while negative values represent equatorward shift at the range margin. (C) Cumulative change in latitudinal extent of species’ range when range margin shift at poleward and equatorward margins is combined.

Passerines’ poleward range margins extended further north through time (0.65 km/decade ± 6.01 SE). At the equatorward margins, the average latitudinal shift was 5.45 km/decade poleward ± 6.86 km SE (Fig. 6A and B). When range movement at both poleward and equatorward margins are considered, species’ breeding ranges have, on average, decreased in their latitudinal extents by 4.80 km/decade ± 10.03 SE (Fig. 6C): Although average poleward shifts were modest, equatorward margin retraction has resulted in overall species’ loss of range extent.

**Figure 7:**
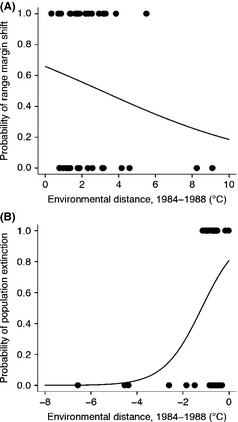
Probability of range margin shift based on local changes in mean breeding season temperature as a function of thermal niche proximity for (A) poleward margin (log likelihood = −22.9, *P* = 0.26) and (B) equatorward margin (log likelihood = −19.8, *P* = 0.008). Populations closer to warm niche limits were more likely to be lost through time, while colonization at the poleward margin was not related to the proximity of those populations to cold niche limits in the early time period. Expectations were based on the direction of climate change (warming or cooling) at occupied sites along range margins.

Geographical range responses among passerines in this study should depend on the proximity of breeding season temperatures at the range margin (either poleward or equatorward) to the coldest or warmest breeding season temperatures encountered anywhere in their breeding ranges. Although we expected poleward range expansion over time to be more likely among species whose poleward populations were nearest the coldest conditions, this was not the case (Fig. 7A; log likelihood = −22.9, *n* = 34, *P*‐value = 0.26). This lack of relationship held when only neotropical migrants were included (Fig. S3a, log likelihood = −17.4, *n* = 26, *P*‐value = 0.28, see Appendix S1). However, population extinctions (and consequent range retraction) (i.e., loss of historical, range margin populations) from species’ equatorward range margins became more likely when these locations were closer to the upper end of species’ realized thermal niche limits (Fig. 7B; log likelihood = −19.8, *n* = 34, *P*‐value = 0.008). This result was consistent also for neotropical species (Fig. S3b, log likelihood = −15.1, *n* = 26, *P*‐value = 0.016). Species bounded by the Gulf of Mexico at their equatorward margin could not be examined as the number of species was low, and these species exhibited little variance in temperature between the equatorward margin and warm thermal niche (<1°C).

Change in mean breeding season temperature had a weak but positive relationship with abundance change at the poleward limit (Fig. S4a; *R* ^2^ = 0.15, *n* = 34, *P*‐value = 0.02). We found no relationship between mean abundance and temperature changes at the cool thermal limit (Fig. S4b; *n* = 34, *P*‐value = 0.3), or at the equatorward margin (Fig. S4c; *n* = 34, *P*‐value = 0.65) or warm (Fig. S4d; *n* = 34, *P*‐value = 0.46) thermal niche limit when the occupied sites from the initial time period were held constant and species’ absences were considered local population extinction. When BBS routes where population extinctions were observed were omitted from the analysis, results were nonsignificant (Fig. S5a–d; *n* = 34, poleward *P*‐value = 0.59; cool *P*‐value = 0.45; equatorward *P*‐value = 0.68; and warm *P*‐value = 0.40; see Appendix S1).

## Discussion

Why and how do species’ ranges shift in response to climate change? There is little doubt that poleward range expansions, now observed widely both taxonomically and geographically (Chen et al. 2011a), are related to rapid, human‐induced climate change. An explanation for such processes is that species’ distributions reflect environmental tolerances, particularly to temperature (Sunday et al. 2012). Climate changes permit pseudo‐experimental tests of range margin determinants (Kerr et al. 2007; Fisher et al. 2010). This hypothesis is supported by observed responses to climate change where poleward margins extend further to the poles, yet fails to explain why equatorward margins have remained relatively stable (or have shifted only slightly) for many species (Chen et al. 2011b). Current hypotheses suggest that as temperatures warm, limitations on poleward range expansion are relaxed and colonists from species’ peripheral populations establish in new areas where populations could not historically persist. Alternatively, peripheral populations may be maintained by colonization pressure from within species’ ranges, so peripheral populations need not be found in areas coinciding with their environmental tolerances. In this case, species’ poleward range boundaries do not directly reflect their environmental tolerances or limits of their thermal niche (Monahan and Tingley 2012) and climate change may cause geographical range responses for reasons other than changing thermal conditions near species’ range boundaries. Geographic responses to climate change along poleward and equatorward range margins in these breeding bird species likely result from different mechanisms. Our results suggest that equatorward range margins are strongly determined by abiotic conditions (specifically average breeding season temperature when this approaches the warm thermal niche limit). This is the opposite expectation from the classic MacArthur (1972) hypothesis, but in line with findings from a wide body of literature investigating limiting factors at equatorward range boundaries (Cahill et al. 2014). For this subset of breeding passerines, equatorward range limits are more strongly related to abiotic conditions than poleward range margins. The latter may respond to entirely different abiotic variables or to composites of climatic conditions.

### Poleward range expansion

Poleward range expansions may be related to warming but do not directly reflect the relaxation of thermal barriers. Breeding bird species demonstrably tolerate temperatures within their ranges that are significantly colder than those observed along their poleward limits during the breeding season, so climate changes to date along those range margins cannot directly facilitate range expansion by relaxing temperature‐related barriers to poleward colonization. The gap between temperatures along species’ poleward range boundaries and the coldest temperatures in which breeding individuals are observed is variable but tends to be substantial in the historical time period (2.55°C ± 1.95°C SD). Yet, temperatures observed along poleward range margins are strongly and linearly related to those observed in the coldest areas of species’ breeding ranges. This gap remains consistent through time, and the slope of the relationship does not differ from 1:1, suggesting that although poleward range margins are not directly limited by breeding season temperature, species are tracking the changing thermal conditions at their poleward margin. Had there been systematic lags (i.e., an incurred climate debt) in rates of range expansion relative to rates of changing breeding season temperature, the gap would have grown larger through time. Breeding birds in North America do not show a trend of climate lag that has been reported in Britain and continental Europe for bird communities (Devictor et al. 2008, 2012). If the offset were driven by differences in radiative heat or other processes not captured through air temperature, we would expect that these processes affect all sites without directional bias. This does not seem to be the case as poleward margin temperatures are always (and at times substantially) warmer than the realized thermal limit. At local scales, which are experienced by passerines, climates are not distributed along a gradient where higher latitudes always have cooler temperatures. Species with poleward range margin populations nearer their lower realized thermal niche limits were not more likely to shift north with warming, a trend that is also consistent within neotropical migrants (see Supplemental Materials). It remains possible that other climatic variables or even composites of climate are more directly related to poleward range shifts; however, the critical distinction remains the observation that abiotic determinants at poleward range margins differ from those at equatorward margins. The question of which populations are sources for poleward range expansion and the cue for these populations to shift remain uncertain, and this area should be a focus for future research.

### Equatorward margin retraction

Population extinction along equatorward range margins is more likely if those populations occupy areas near the upper thermal limits of the species’ realized niche. Along species’ equatorward range edges, warming temperatures may exceed species’ physiological limits, even after accounting for flexibility derived from behavioral thermoregulation, leading to population losses. Population extinction due to climate change can occur rapidly and lead to range retraction even over the relatively short time period of this study. These effects likely depend on the fact that temperatures observed along equatorward range boundaries tend to approximate the hottest temperatures observed within these bird species’ breeding ranges. Moreover, the historical temperatures observed among equatorward populations relative to the warmest areas within species’ ranges are strongly related through time. The temperature difference between range margins and thermal niche limits is smaller at species’ equatorward range boundaries, and that gap increased slightly through time along equatorward range margins, an effect driven by equatorward margin retraction. In other words, even if the southernmost populations of these passerines are not found in areas that are the warmest the species experiences in its breeding range, warming trends since 1984–1988 have brought equatorward populations closer to those limits. We observe increased population extinction risk at equatorward margins as species’ thermal limits approach. Local extinctions along equatorward routes increase the distance separation between realized niche limits and equatorward margin temperatures in later time periods.

### Differences between poleward and equatorward response

There is no doubt that bird species’ range limits, whether they are resident or migratory, reflects an array of environmental and biotic interactions (Melles et al. 2011; La Sorte & Jetz 2012), which may complicate expectations of range shifts among these species. Many studies report bird species’ range shifts in the direction expected from climate change (Thomas and Lennon 1999; Parmesan and Yohe 2003; Brommer 2004). Among North American breeding birds in this study, poleward range limits do not directly reflect these species’ realized thermal limit, yet the thermal distance between realized thermal limits and poleward margin temperatures has been maintained through time. Warming along poleward margins cannot consequently generate either pressure or opportunity for colonization of unoccupied areas through mechanisms reflecting changes in direct thermal suitability. Species’ physiological tolerance may be less constrained at poleward range margins than at southern range margins because traits relating to cold tolerance may vary more among lineages, while traits relating to heat tolerance exhibit greater niche conservatism (Araújo et al. 2013). Although range margin shifts, as reported in the literature, are more dramatic at poleward margins (Parmesan and Yohe 2003), the mechanism for these shifts are unlikely to be due to direct cold limitation at these locations. Other mechanisms that consider interaction between range expansion and colonization from within species’ ranges may be necessary to explain species’ poleward margin geographical responses to climate change. We further hypothesize that observed differences in response between poleward and equatorward margins can also be explained if poleward limits are further from the true fundamental thermal niche limits than equatorward portions of the range. This may arise due to other limiting factors such as temperature variability or resource availability. Localized differences in climate change lead to an overall loss in range extent for the 34 species included in this study without a corresponding loss of thermal placement at range margins; it is evident that geographic response at poleward and equatorward range margins can lead to significant climate impact on range extent without climate debt, or systematic lags in response to climate change, being observed.

### Effects of extreme heat events

Species’ geographic range margins are not in equilibrium with realized thermal limits; thus, geographic responses to date may underestimate the true cost of climate change on species’ distributions. If warming temperatures shift climatic niche space closer to species’ range margins, peripheral populations will become more vulnerable to extreme heat events as well as the more subtle or indirect effects of warming. If so, range margins must be directly mediated by thermal tolerance. Heat waves and/or drought can cause mortality rates of both adults and juveniles to rise (Martin et al. 2007; Robinson et al. 2007; McKechnie and Wolf 2010; Cox et al. 2013) and bird species’ richness and abundance to decline (Albright et al. 2010, 2011). This mechanism is likely to explain current range losses from breeding birds’ equatorward range margins. Adult birds, many of which are neotropical migrants that experience warmer conditions in their overwintering grounds, are likely less affected by breeding season warming trends than juveniles, but more research into the effects of temperature extremes arising from extreme weather events on bird mortality and population viability is necessary to test this mechanism in areas where populations are confronted with elevated extinction risk related to approaching thermal niche limits. Detecting these effects reliably across many species’ geographical ranges will require expanded population‐level observations that the BBS was not designed to provide.

### Data limitations

We have adopted conservative species and BBS route selection criteria that limit the potential impacts of variation in sampling intensity on measurements of extinction risk or range expansion. Limiting analyses to well‐sampled species whose entire breeding ranges were within the most intensively sampled areas of North America reduced the number of species available for analyses predicting range responses, but meant that we could test for shifts along both poleward and equatorward range limits relative to breeding range thermal niche limits. Given sample size limitations imposed by such conservative species’ selection criteria, we anticipated that the most significant limitations of this study would reflect problems of statistical power and consequent failures to detect significant biological trends. Results reported may be conservative. In the future, expanded BBS results may permit higher resolution analysis of temperature trends that may improve precision around estimations of species’ geographical thermal limits (sensu Jiguet et al. 2006), but it seems unlikely that this will qualitatively alter our findings. We have not attempted to interpret changes in abundance observations among these routes, which may not be comparable across the geographical extent of North America or through time given observer and environmental variation among BBS routes (Sauer et al. 2004; see Appendix S1 for more information). Species‐specific rates of range margin shift reflect locality‐ and species‐specific factors (Chen et al. 2011a), and climate‐related shifts may not be sufficiently large to be distinct from such effects.

## Conclusion

Recent climate changes exert a measurably large impact on breeding bird species’ distributions across well‐sampled regions of North America. These species’ equatorward range margins appear to have shifted in response to these abiotic changes. The observed difference in response to thermal limit proximity at equatorward and poleward margins lend support to the argument that thermal limits are more constrained at equatorward margins (Araújo et al. 2013), and suggest that breeding distributions are located closer to fundamental thermal limits at the hot end of the distribution. Species’ poleward ranges likely did not expand with warming temperatures because they are not limited directly by ambient temperature in the breeding season (see Sax et al. 2013). We speculate that poleward populations may be maintained through interactions with populations nearer the cores of species’ ranges, which may be closer to thermally optimal areas. Poleward range expansions are facultative and only represent a negative impact of warming to the extent that species are not shifting rapidly enough to avoid incurring climate debts (Devictor et al. 2012; La Sorte and Jetz 2012). Population extinctions in areas that have warmed beyond species’ historical thermal limits, conversely, are a clear, rapid, and decisively negative impact of warming. Yet, many species have retracting equatorward ranges and expanding poleward ranges that result in net range loss among many passerines included here (Fig. 6C). Climate changes observed to date may exert a negative impact on such species directly through the breadth of their geographic ranges. Understanding the specific causes of climate‐driven range dynamics at poleward and equatorward range margins is critical if impacts on populations and species are to be successfully mitigated.

## Conflict of Interest

None declared.

## Supporting information


**Figure S1.** Relationship between mean spring temperature at the realized niche margin (calculated as the 10 coldest/warmest routes with species occurrence during 1984–1988) and mean spring temperature at the range margin expected to correspond to the niche edge (calculated as the 10 poleward/equatorward routes with species occurrence during 1984–1988) at the a) cool edge and b) warm edge for neotropical migrants (*n* = 26) and c) cool edge and d) warm edge for species bounded by the Gulf of Mexico at their equatorward margin (*n* = 21). Circular data points represent species that are shifting as expected given temperature changes.
**Figure S2.** Temporal relationship in environmental distance calculated as the difference in mean spring temperature (°C) separating the thermal niche boundary and range margin in 1984–1988 and 2002–2006 for a) the poleward margin and cool niche boundary, and b) the equatorward margin and warm niche boundary for neotropical migrants (*n* = 26), and c) the poleward margin and cool niche boundary, and d) the equatorward margin and warm niche boundary for species bounded by the Gulf of Mexico (*n* = 21). Circular data points represent species that are shifting as expected given temperature changes.
**Figure S3.** Probability of range margin shift based on local changes in mean spring temperature as a function of thermal niche proximity for a) poleward margin (log‐likelihood = −21.36277, *P* = 0.21), and b) equatorward margin (log‐likelihood = −20.14979, *P* = 0.059).
**Figure S4.** Mean abundance change with change in temperature from 1984–1988 to 2002–2006 for a) cool thermal limit, b) warm thermal limit, and c) poleward, and d) equatorward margin.
**Figure S5.** Mean abundance change with change in temperature from 1984–1988 to 2002–2006 for a) cool thermal limit, b) warm thermal limit, c) poleward margin, and d) equatorward margin.

**Appendix S1.** List of passerine species.
