# Supplementary material for: Temperature‐related geographical shifts among passerines: contrasting processes along poleward and equatorward range margins
Source: Ecol Evol. 2015 Oct 20;5(22):5162–76. doi: 10.1002/ece3.1683 (PMC6102530; doi:10.1002/ece3.1683)
Supplement: Supplementary file 1 — Figure S1. Relationship between mean spring temperature at the realized niche margin (calculated as the 10 coldest/warmest routes with species occurrence during 1984–1988) and mean spring temperature at the range margin expected to correspond to the niche edge (calculated as the 10 poleward/equatorward routes with species occurrence during 1984–1988) at the a) cool edge and b) warm edge for neotropical migrants (n = 26) and c) cool edge and d) warm edge for species bounded by the Gulf of Mexico at their equatorward margin (n = 21). Circular data points represent species that are shifting as expected given temperature changes. Figure S2. Temporal relationship in environmental distance calculated as the difference in mean spring temperature (°C) separating the thermal niche boundary and range margin in 1984–1988 and 2002–2006 for a) the poleward margin and cool niche boundary, and b) the equatorward margin and warm niche boundary for neotropical migrants (n = 26), and c) the poleward margin and cool niche boundary, and d) the equatorward margin and warm niche boundary for species bounded by the Gulf of Mexico (n = 21). Circular data points represent species that are shifting as expected given temperature changes. Figure S3. Probability of range margin shift based on local changes in mean spring temperature as a function of thermal niche proximity for a) poleward margin (log‐likelihood = −21.36277, P = 0.21), and b) equatorward margin (log‐likelihood = −20.14979, P = 0.059). Figure S4. Mean abundance change with change in temperature from 1984–1988 to 2002–2006 for a) cool thermal limit, b) warm thermal limit, and c) poleward, and d) equatorward margin. Figure S5. Mean abundance change with change in temperature from 1984–1988 to 2002–2006 for a) cool thermal limit, b) warm thermal limit, c) poleward margin, and d) equatorward margin. [file ECE3-5-5162-s001.pdf]

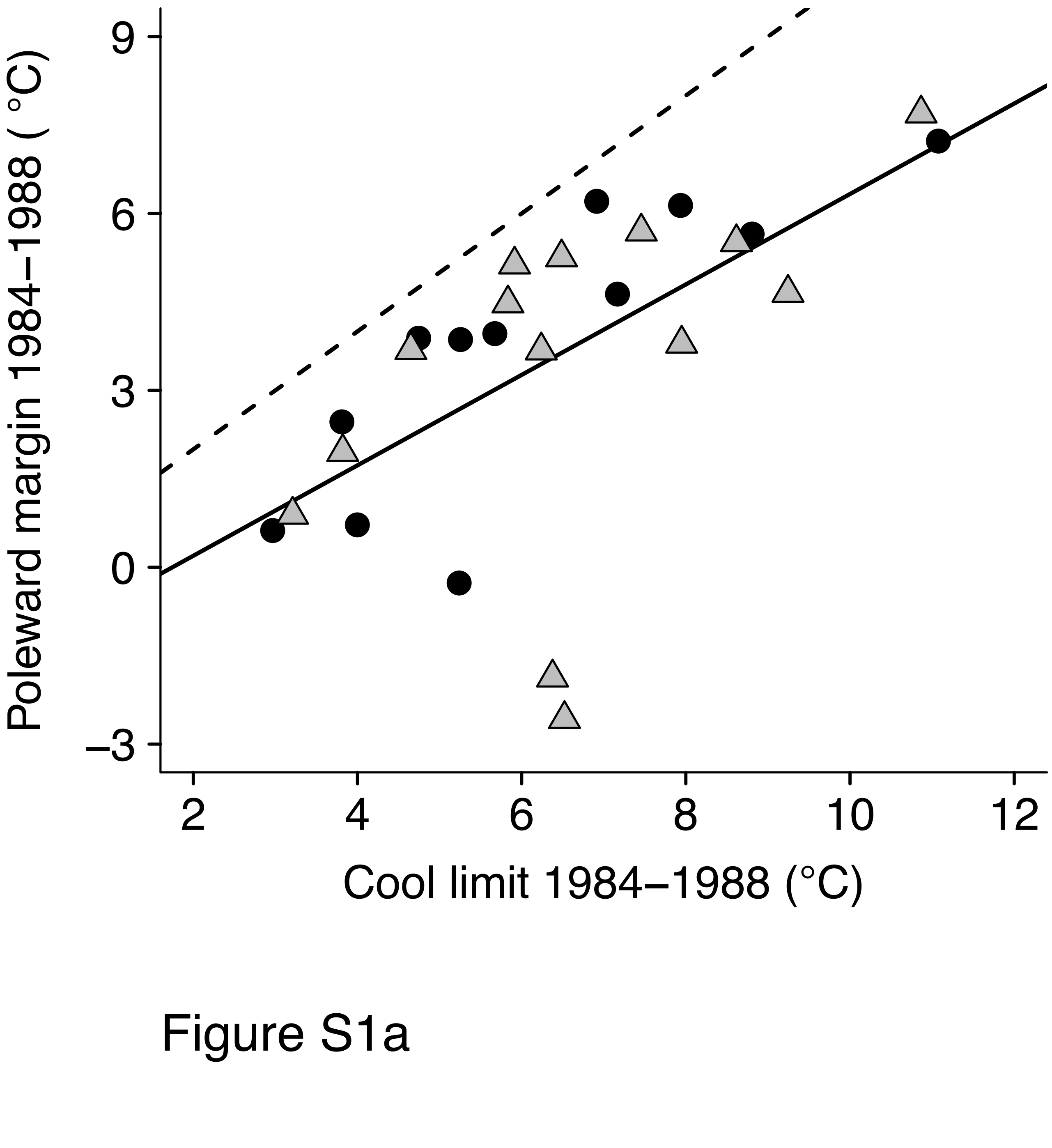
9
6
3
0
–3
2
4
6
8
10
12
Poleward margin 1984–1988 (°C)
Cool limit 1984–1988 (°C)

Figure S1a

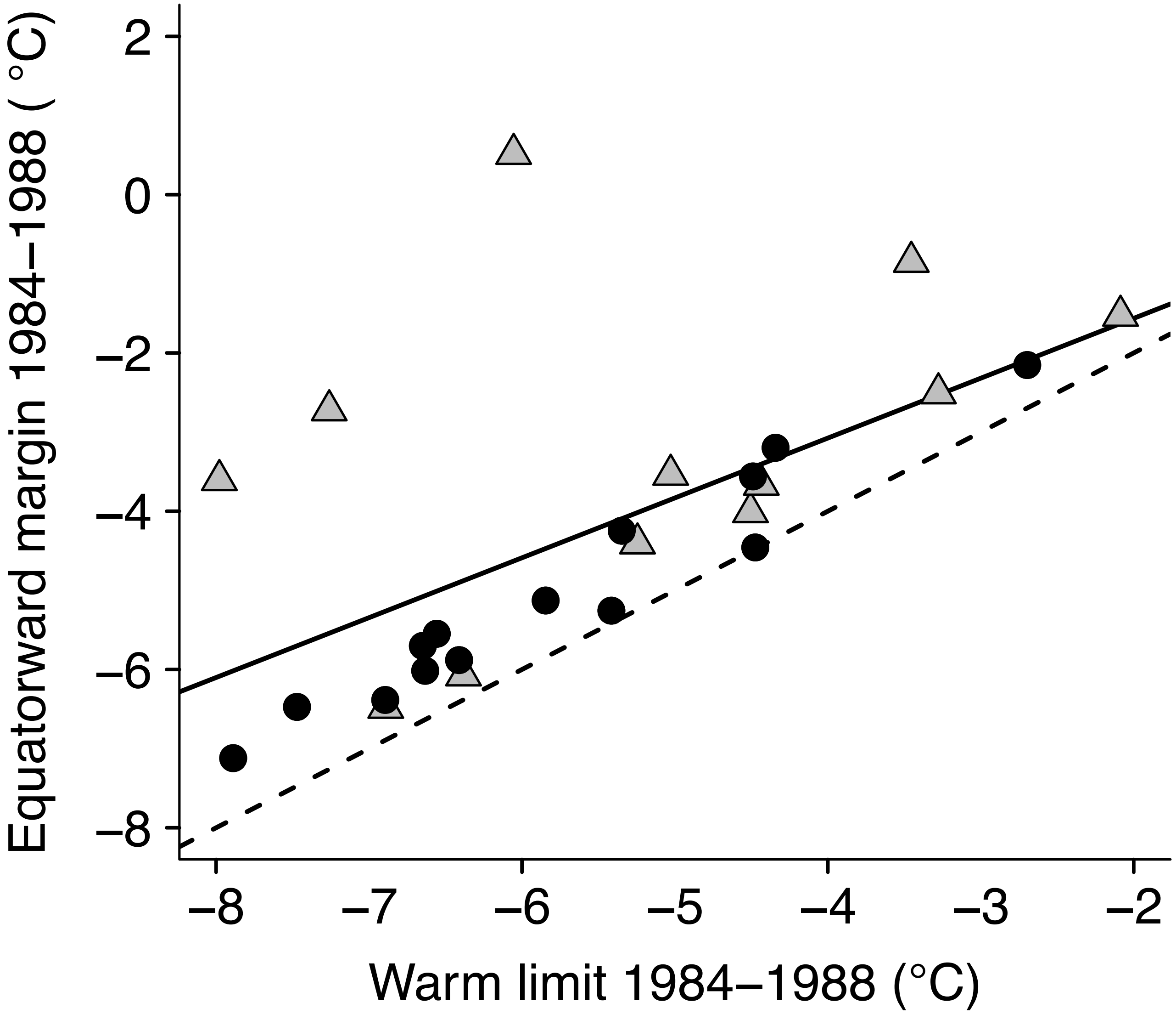
Equatorward margin 1984–1988 (°C)
2
0
−2
−4
−6
−8
−8
−7
−6
−5
−4
−3
−2
Warm limit 1984–1988 (°C)

Figure S1b

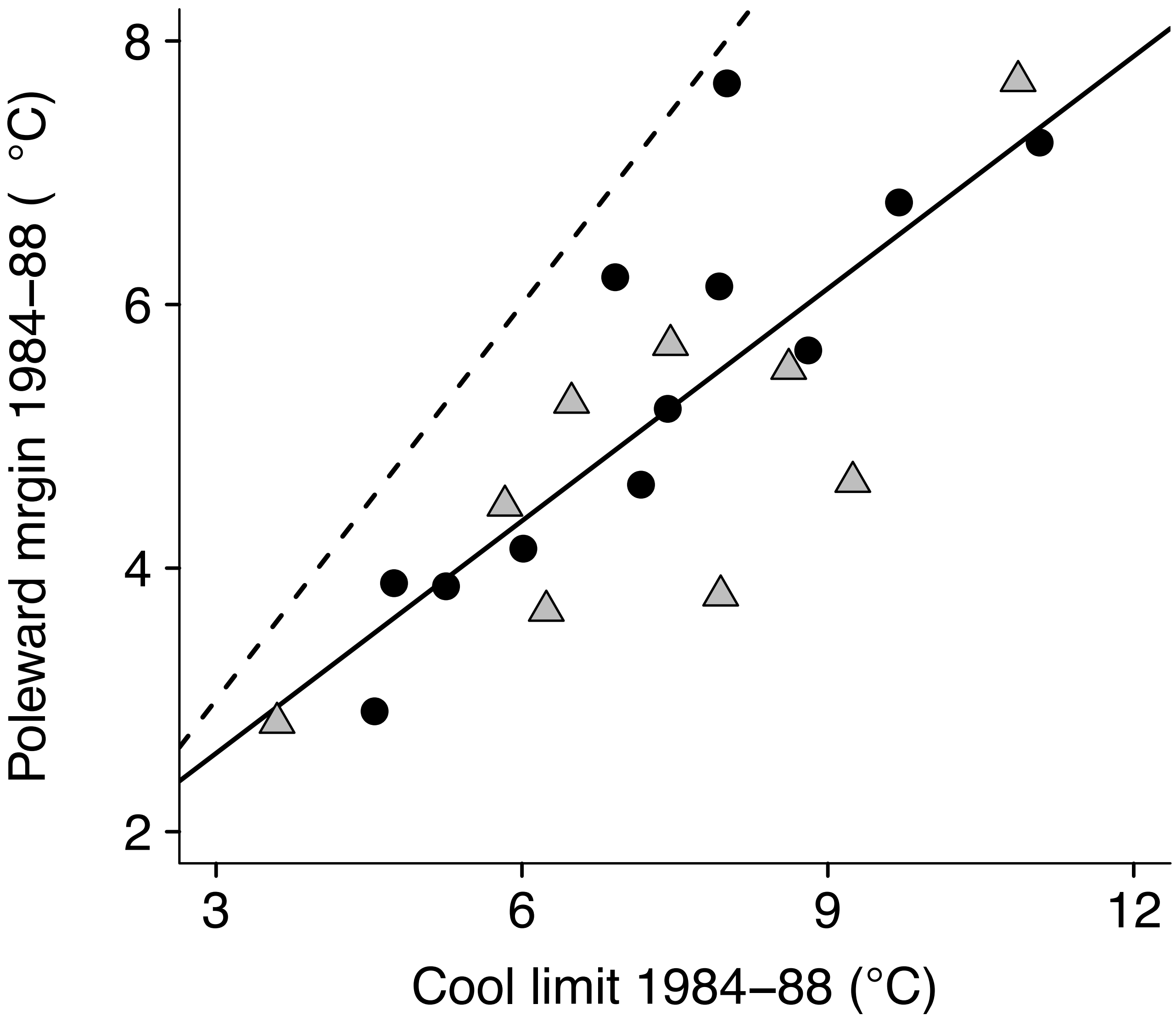

Figure S1c

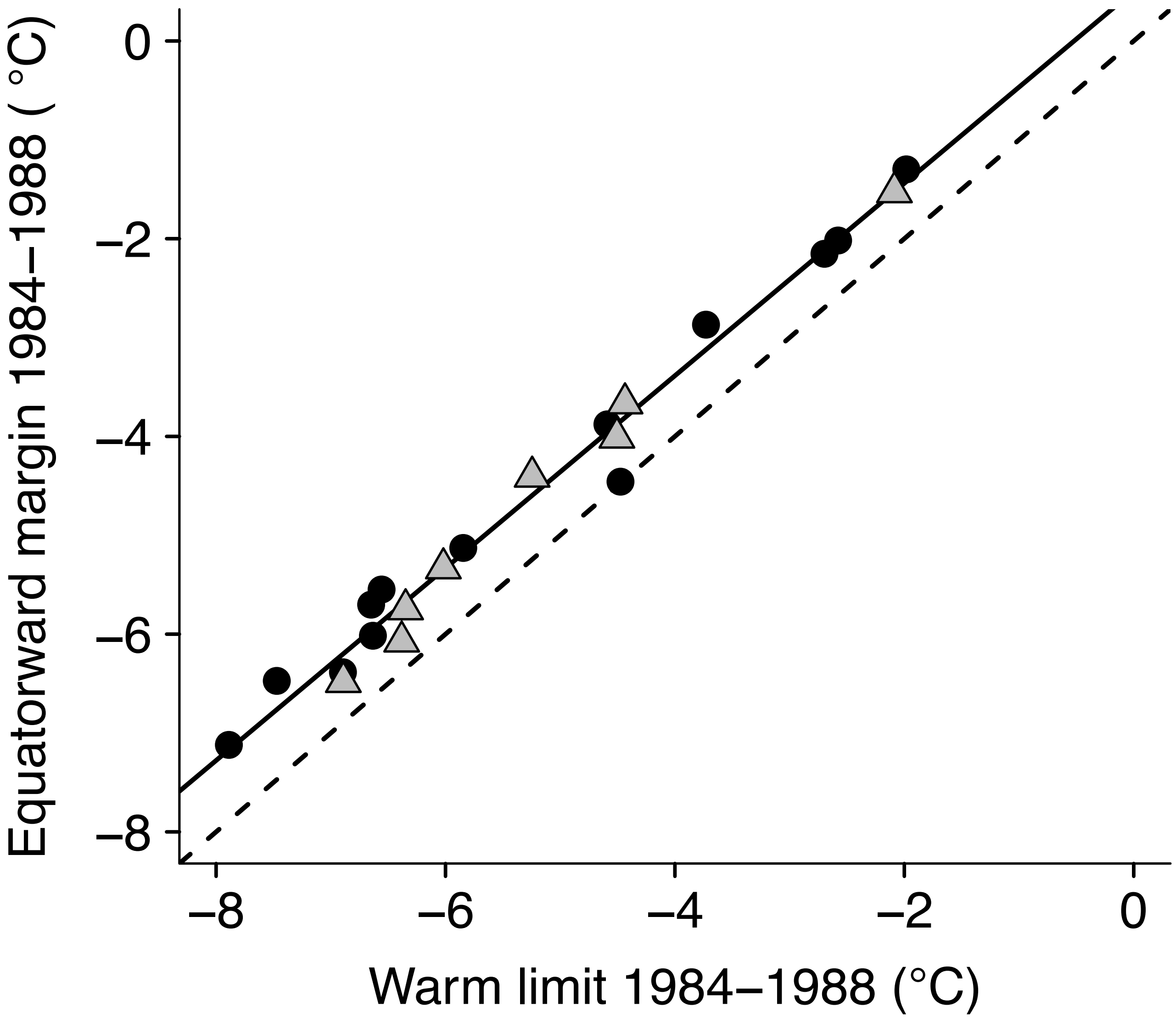
0
−2
−4
−6
−8
Equatorward margin 1984–1988 (°C)
−8
−6
−4
−2
0
Warm limit 1984–1988 (°C)

Figure S1d

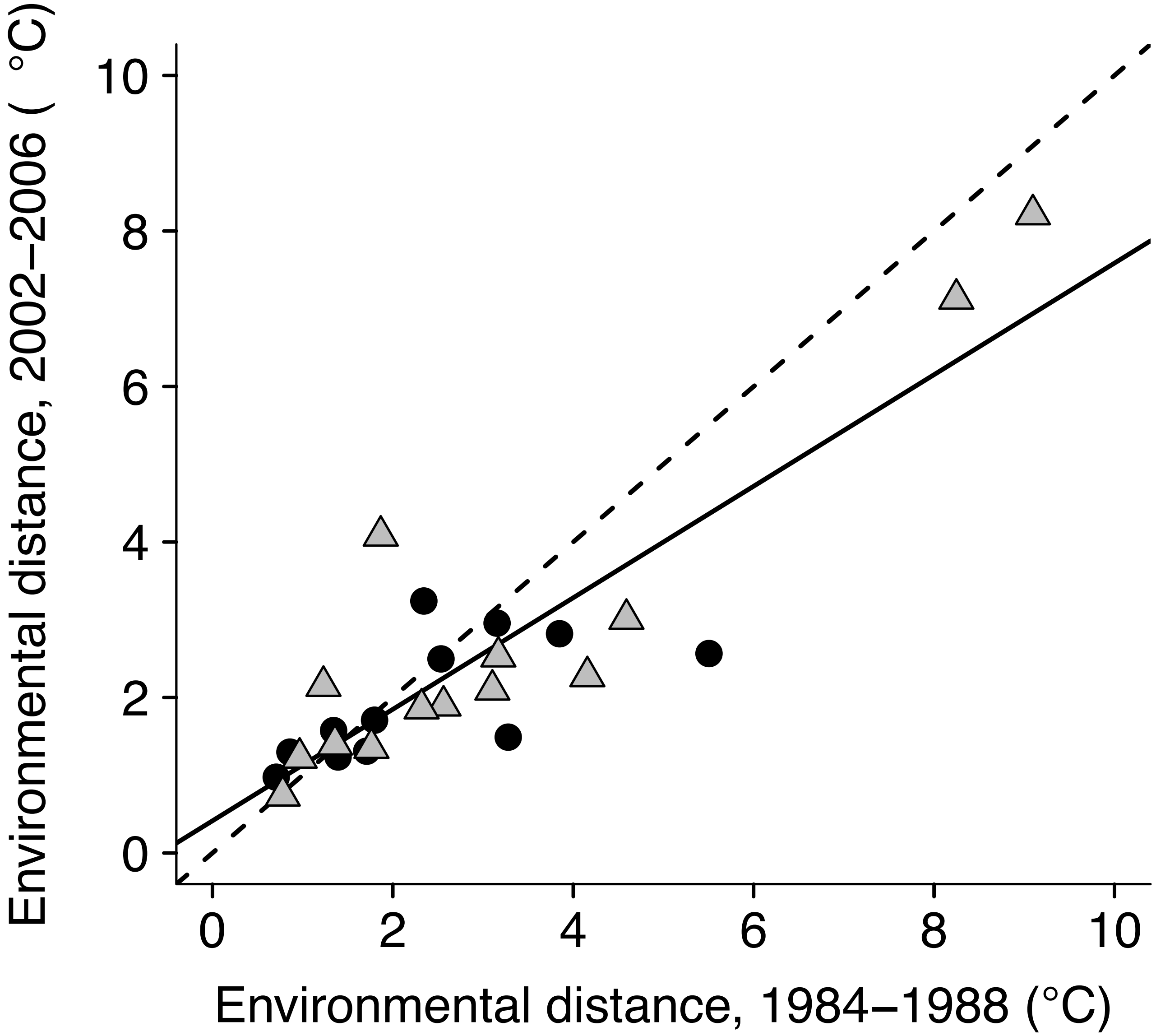

Environmental distance, 2002–2006 (°C)
0
2
4
6
8
10
Environmental distance, 1984–1988 (°C)

Figure S2a

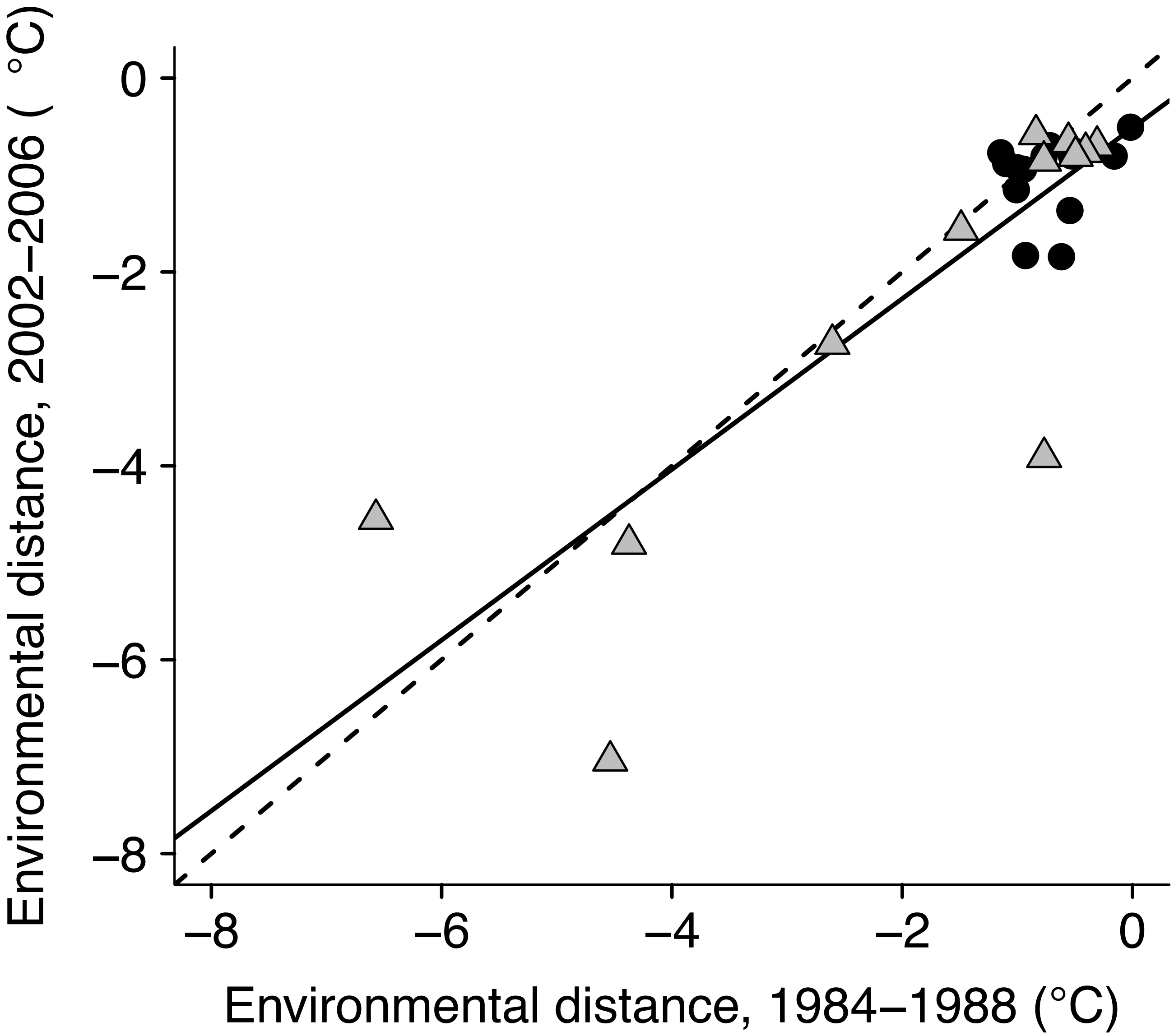
0
−2
−4
−6
−8
Environmental distance, 2002−2006 (°C)
−8
−6
−4
−2
0
Environmental distance, 1984−1988 (°C)

Figure S2b

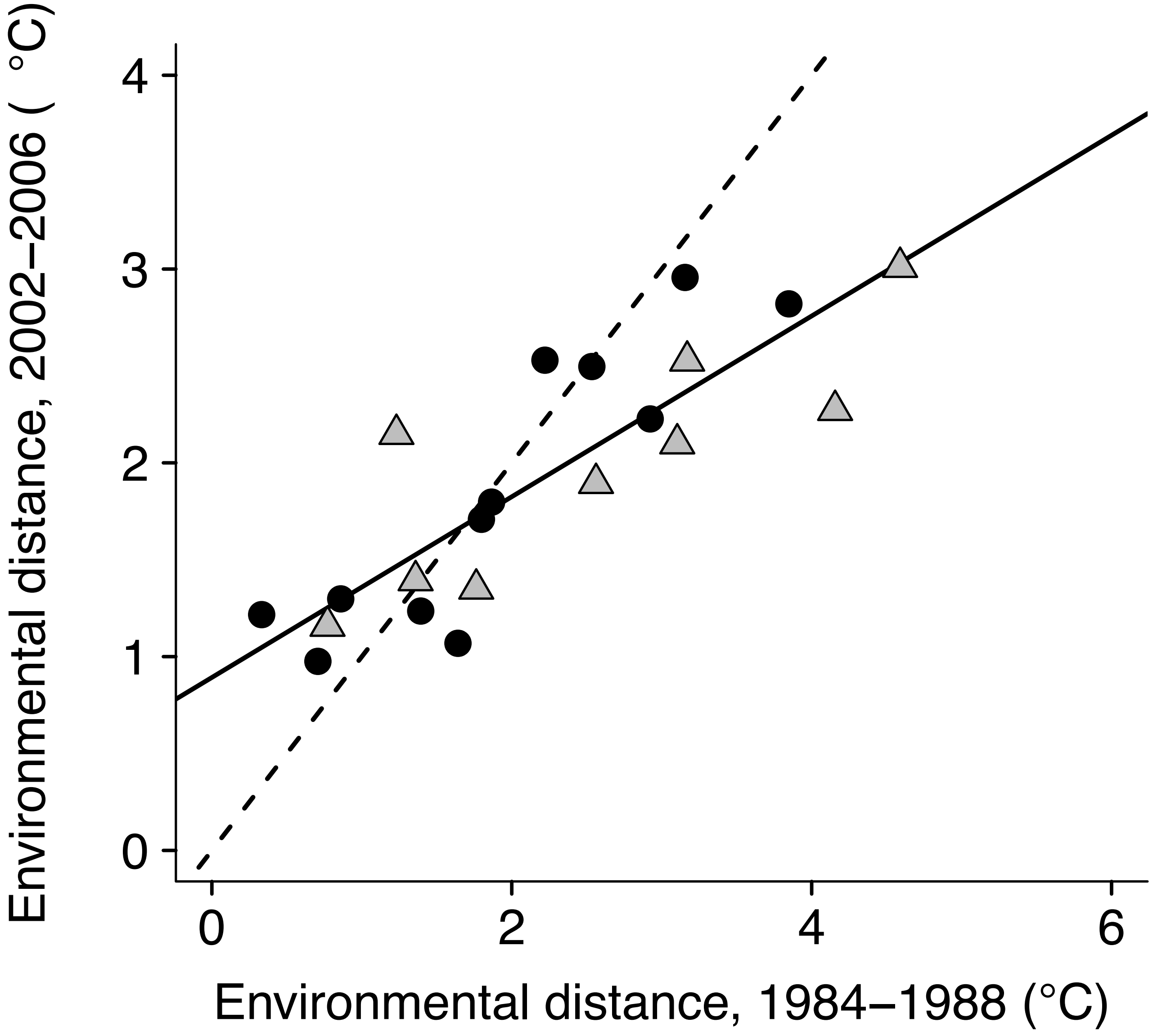
Environmental distance, 2002–2006 (°C)
4
3
2
1
0
0
2
4
6
Environmental distance, 1984–1988 (°C)

Figure S2c

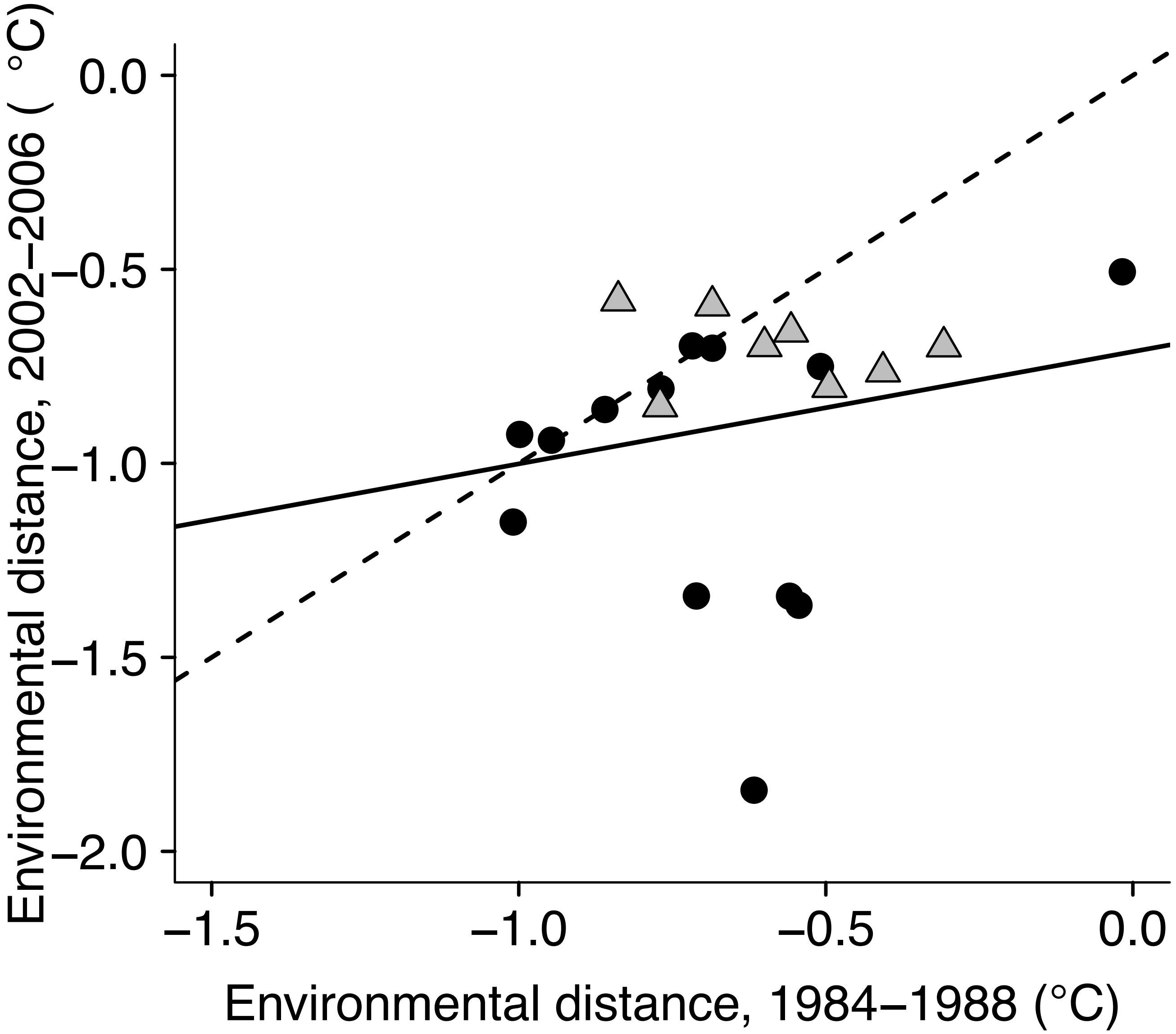
Environmental distance, 2002−2006 (°C)
0.0
−0.5
−1.0
−1.5
−2.0
−1.5
−1.0
−0.5
0.0
Environmental distance, 1984−1988 (°C)

Figure S2d

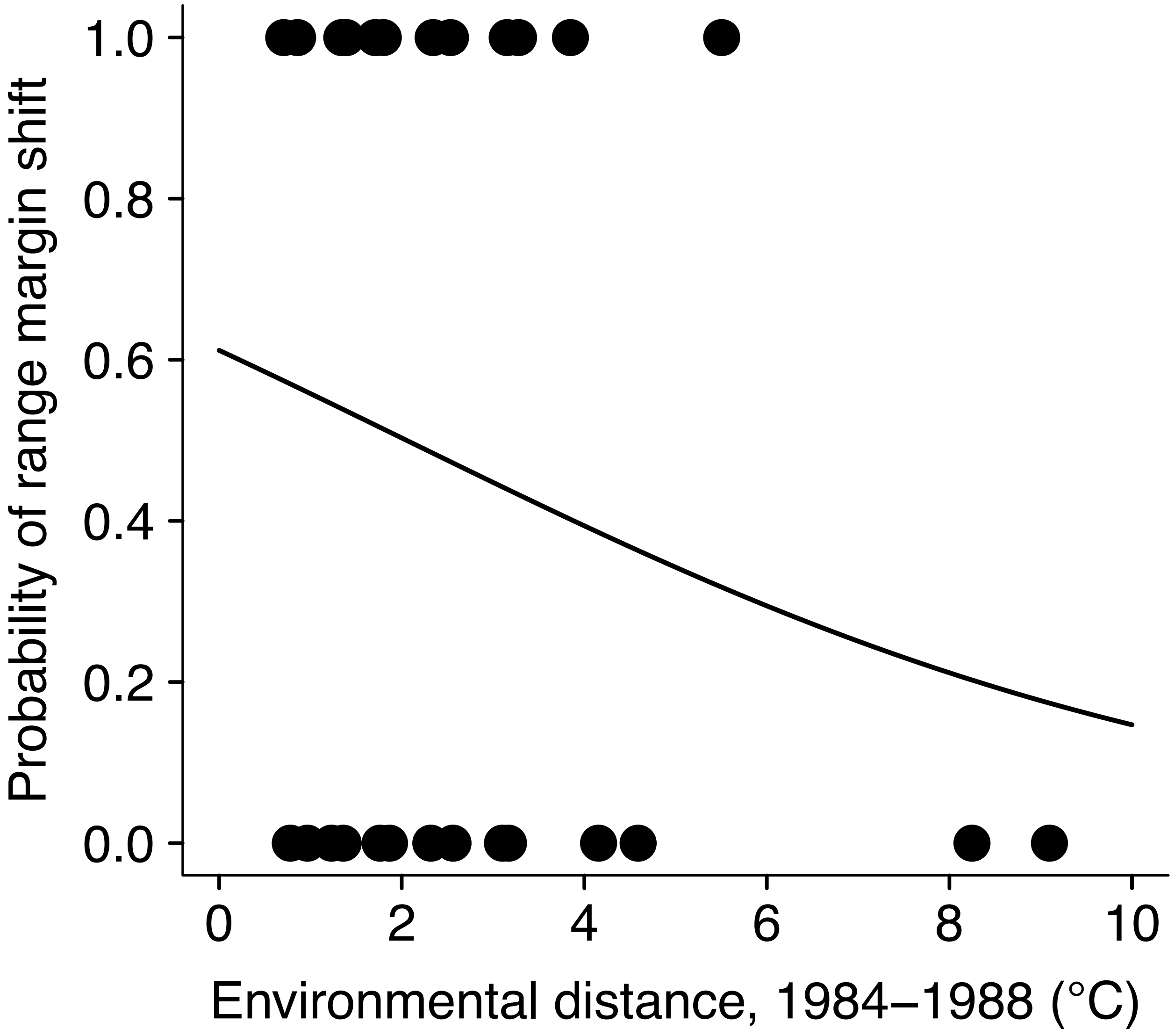
Probability of range margin shift
1.0
0.8
0.6
0.4
0.2
0.0
0
2
4
6
8
10
Environmental distance, 1984–1988 (°C)

Figure S3a

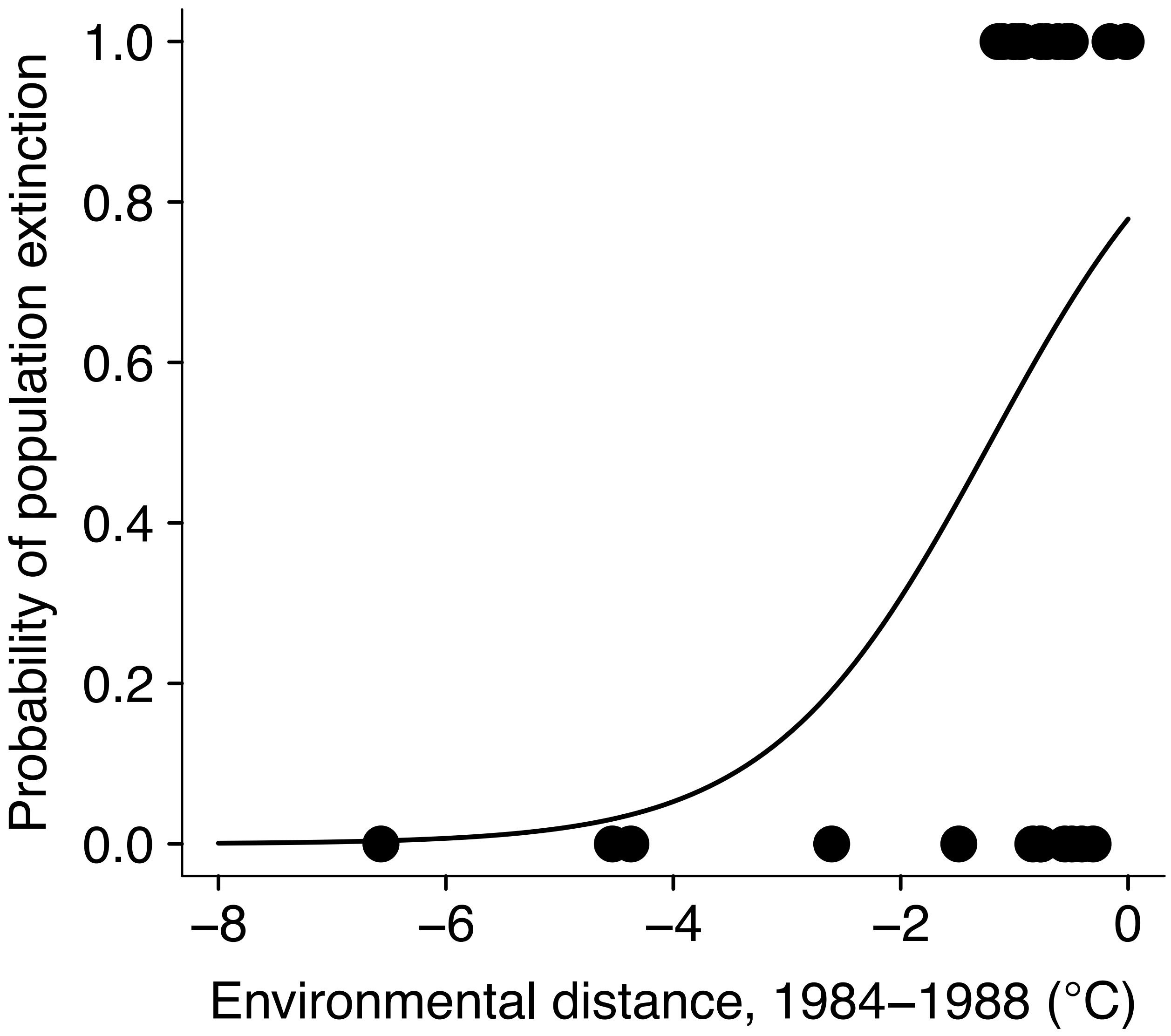

Probability of population extinction
1.0
0.8
0.6
0.4
0.2
0.0
−8
−6
−4
−2
0
Environmental distance, 1984−1988 (°C)

Figure S3b

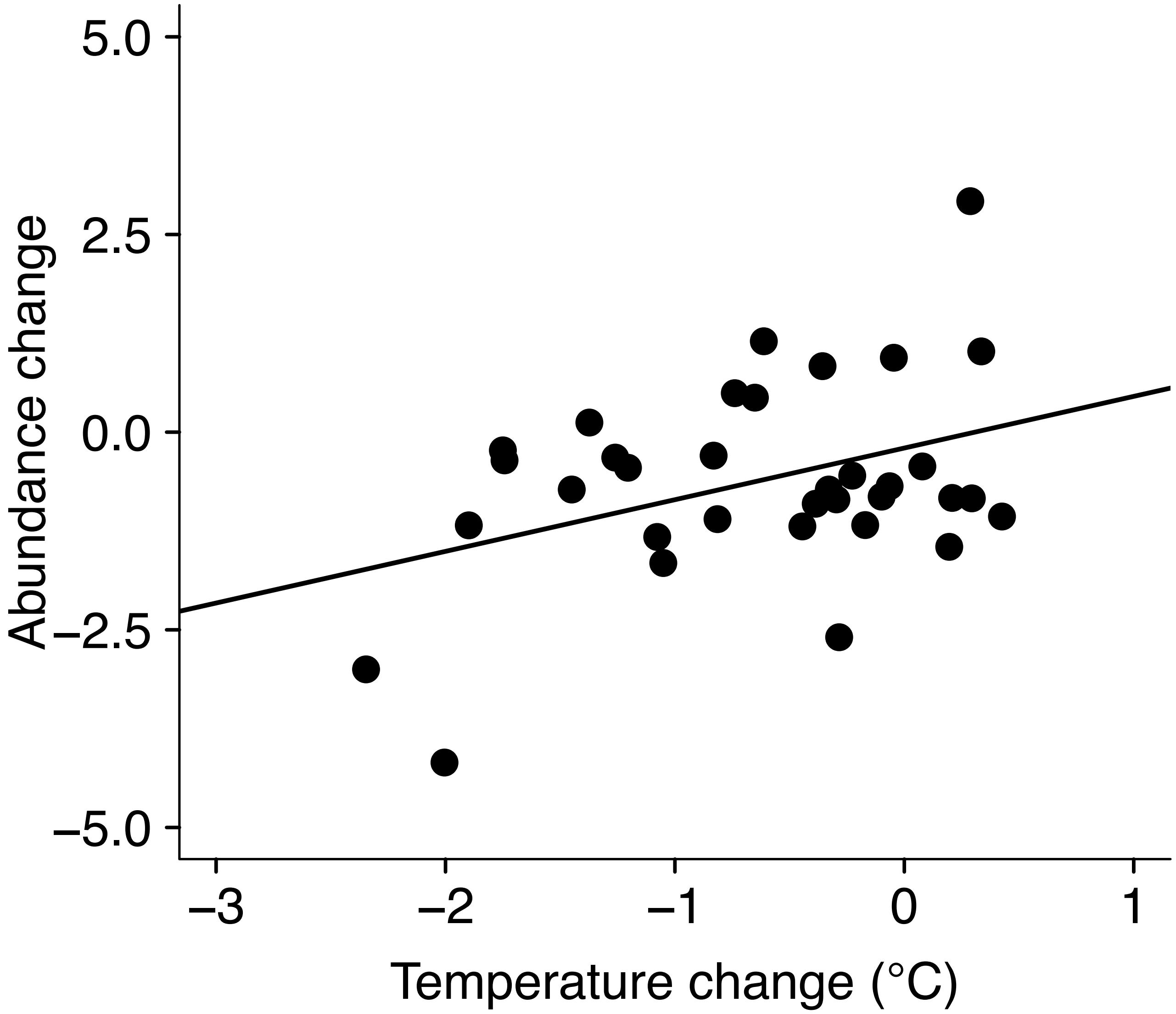

Figure S4a

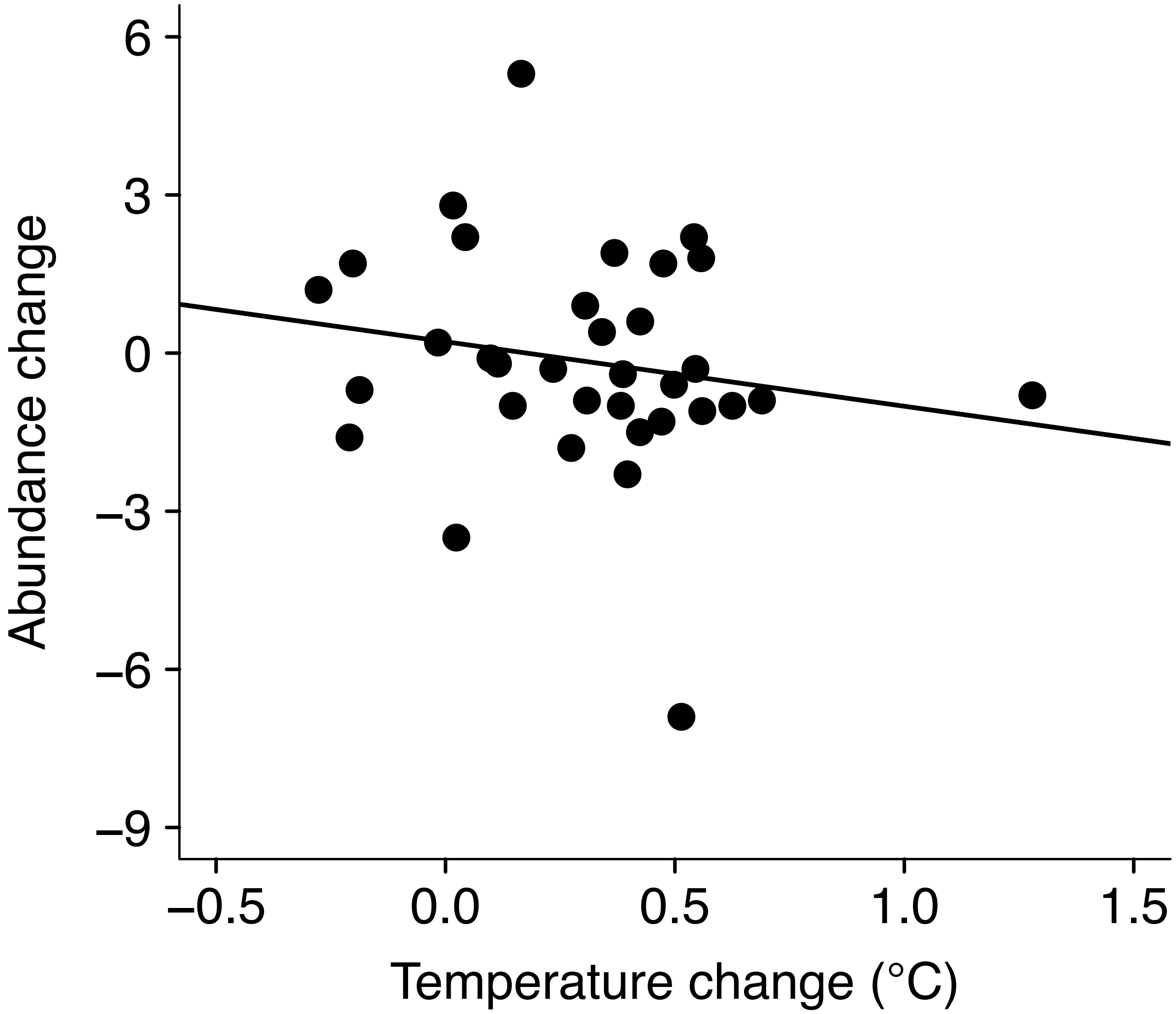

Figure S4b

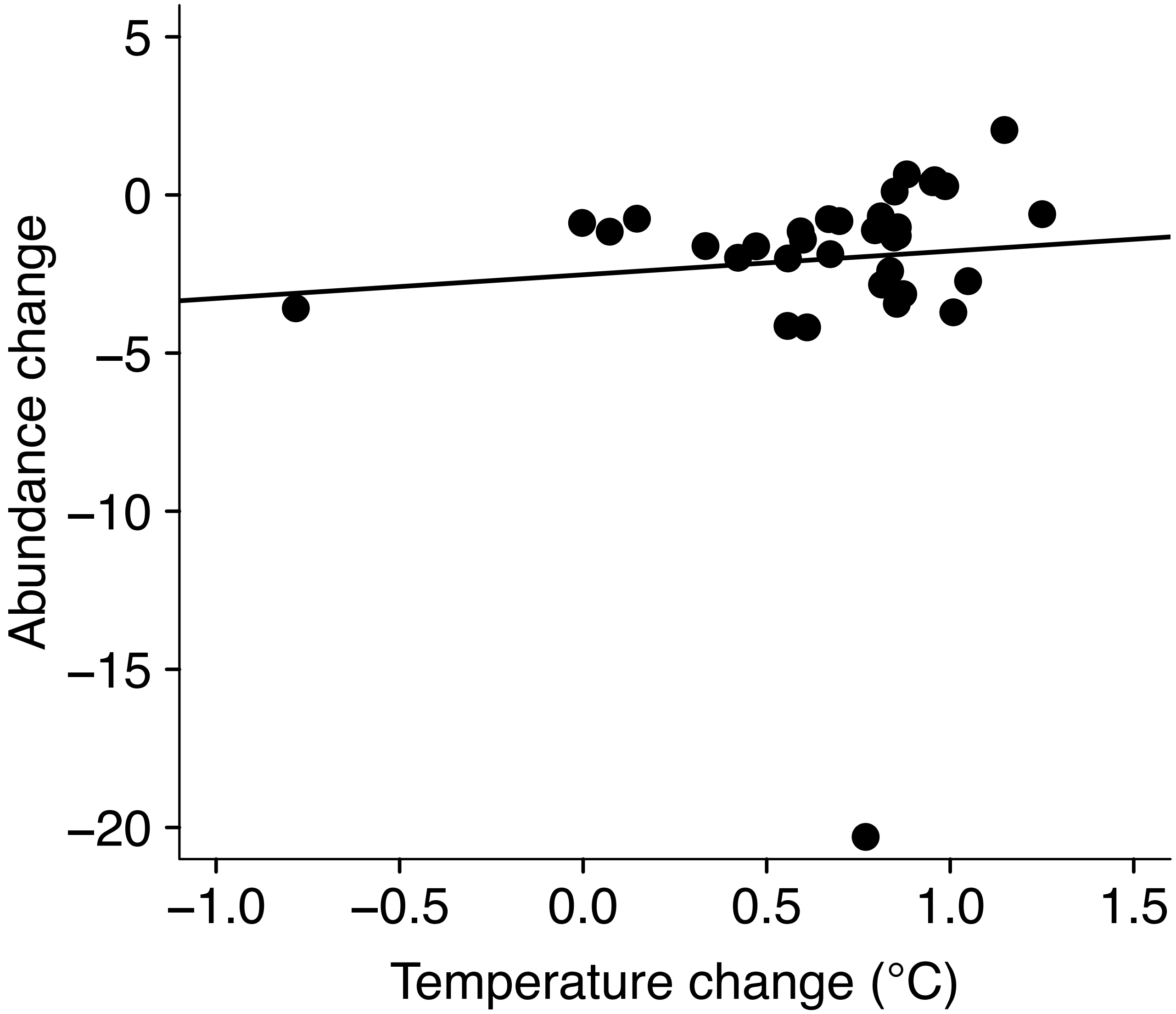
5
0
−5
−10
−15
−20
Abundance change
−1.0
−0.5
0.0
0.5
1.0
1.5
Temperature change (°C)

Figure S4c

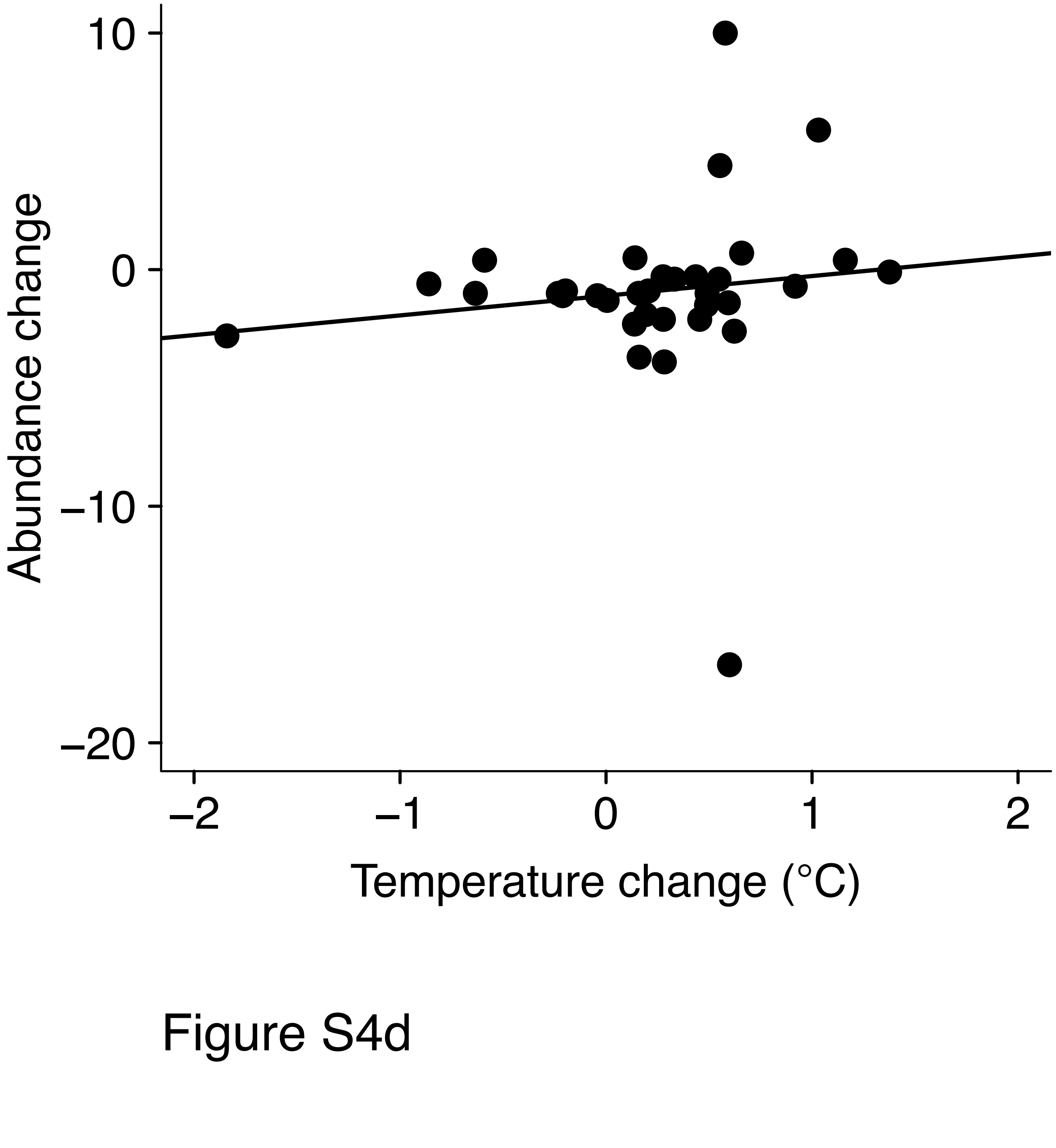

Figure S4d

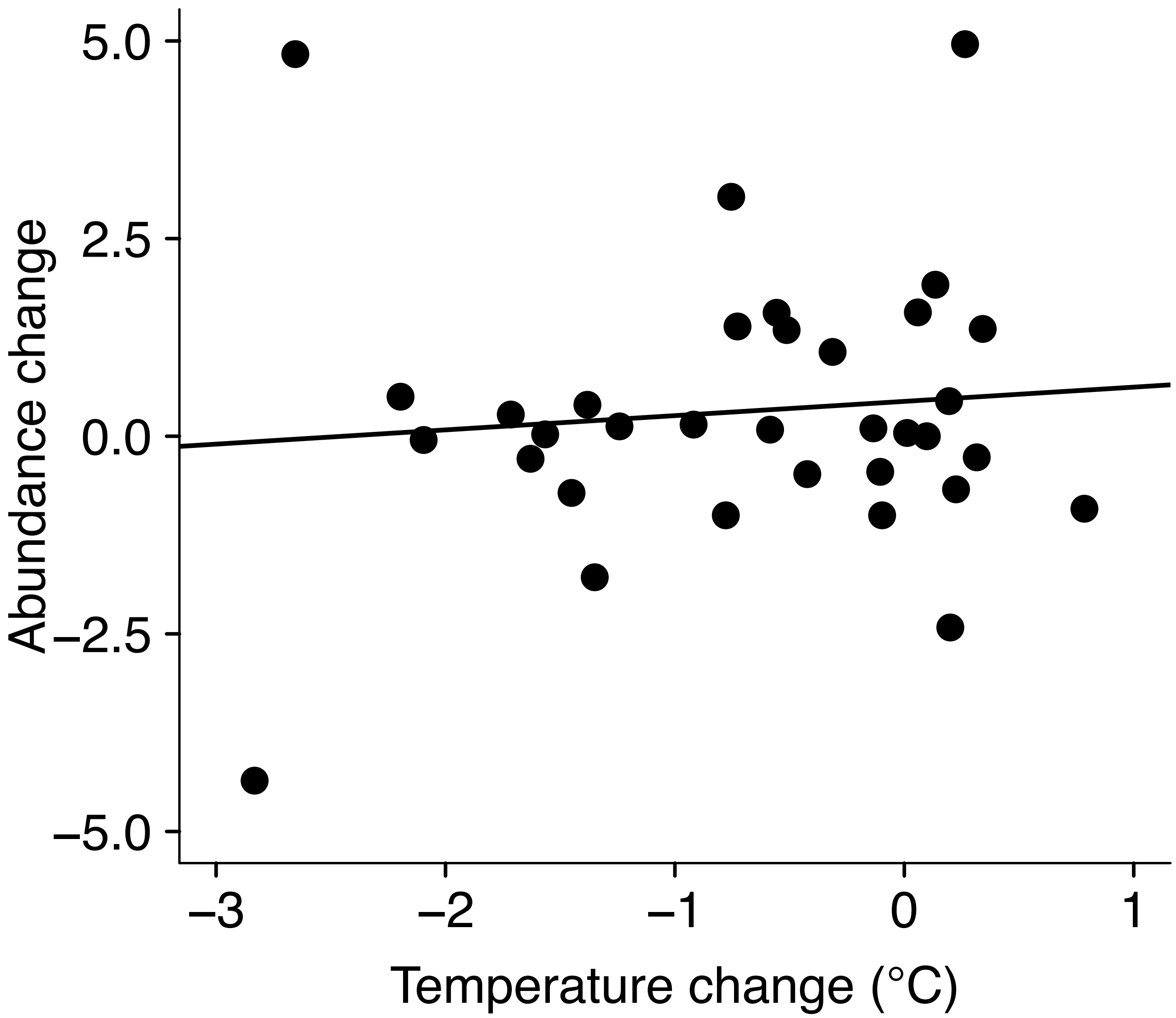

Figure S5a

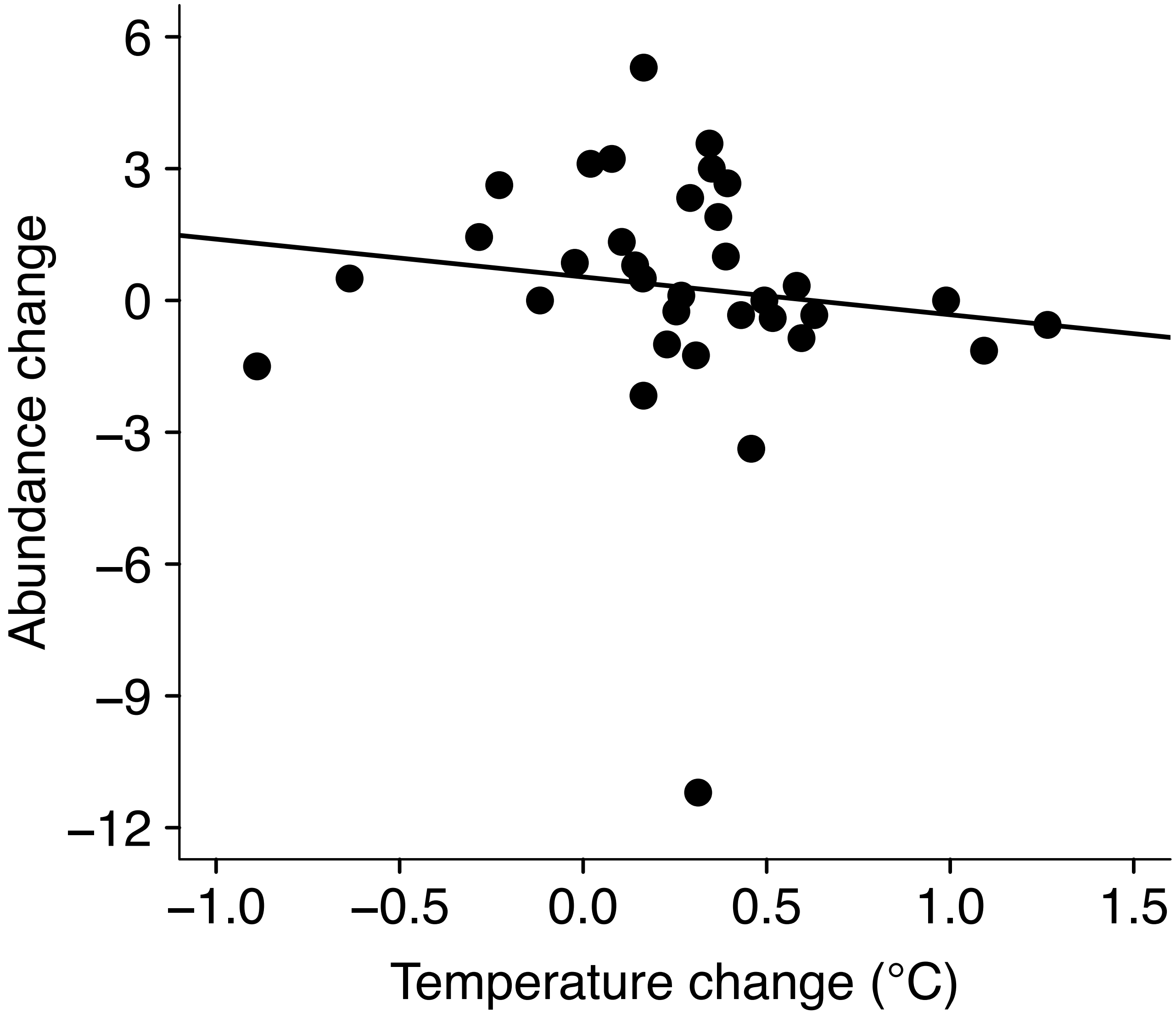
6
3
0
−3
−6
−9
−12
Abundance change
−1.0
−0.5
0.0
0.5
1.0
1.5
Temperature change (°C)

Figure S5b

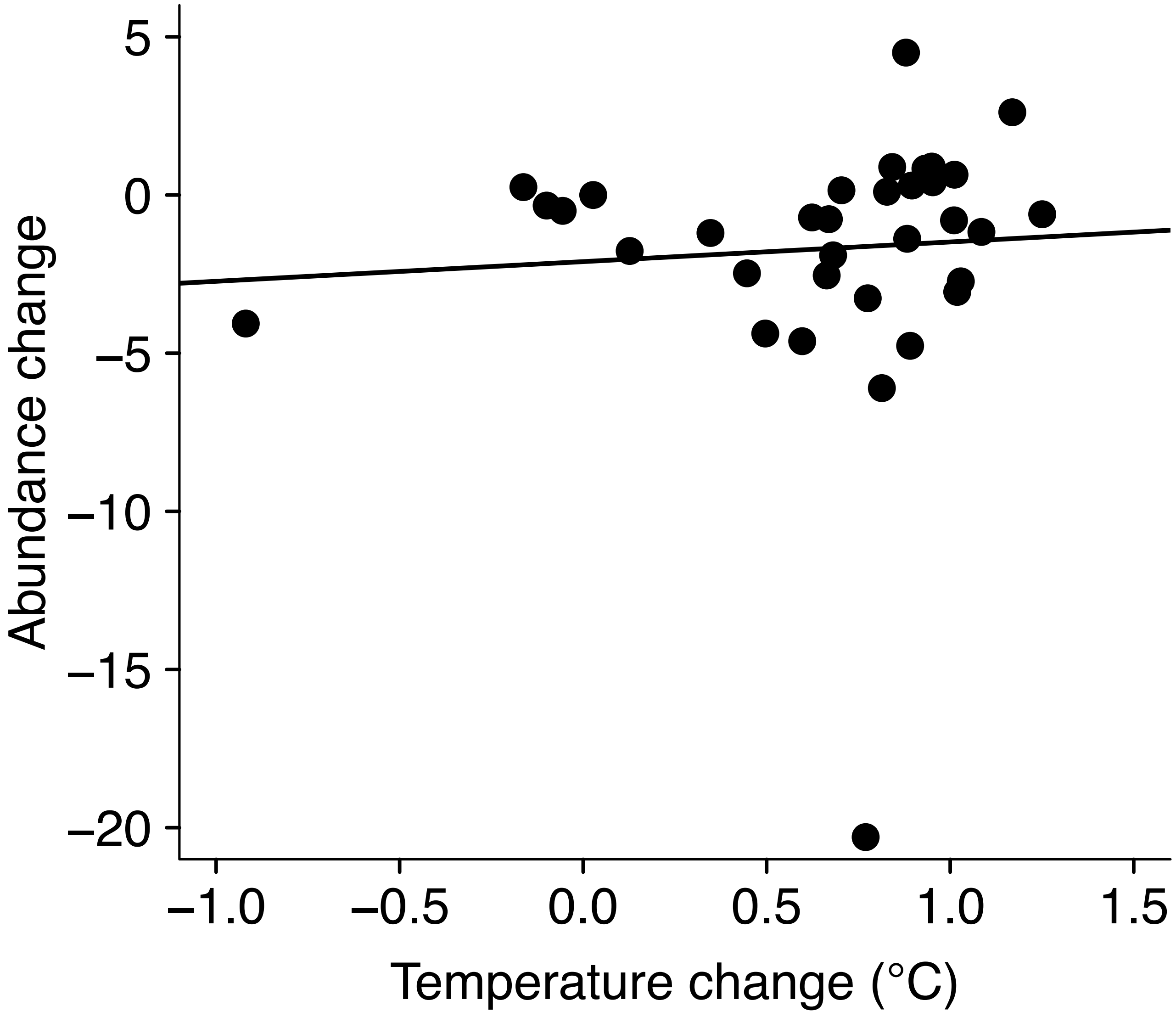
5
0
−5
−10
−15
−20
Abundance change
−1.0
−0.5
0.0
0.5
1.0
1.5
Temperature change (°C)

Figure S5c

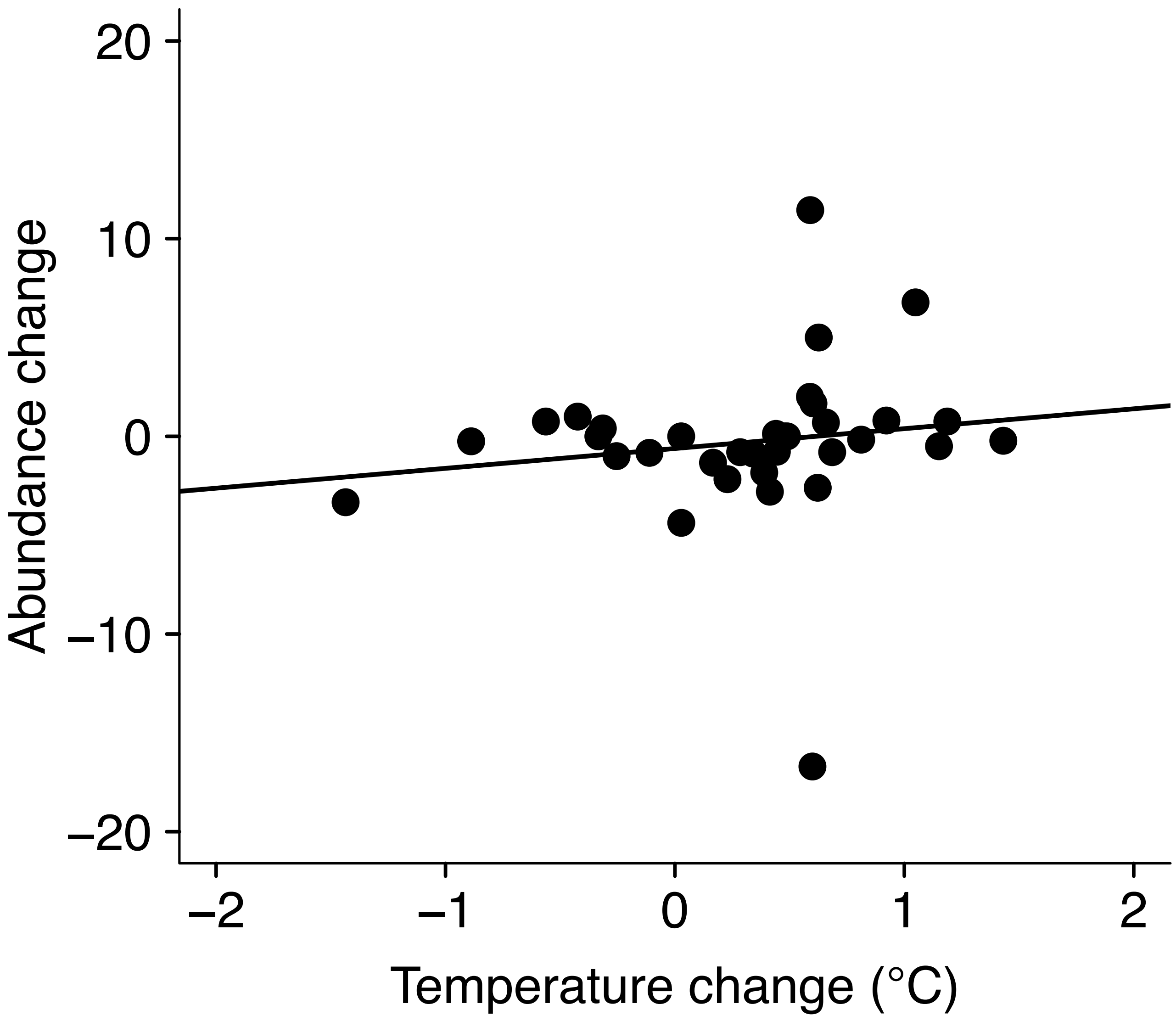

Figure S5d
