# Supplementary material for: Temperature‐related geographical shifts among passerines: contrasting processes along poleward and equatorward range margins
Source: Ecol Evol. 2015 Oct 20;5(22):5162–76. doi: 10.1002/ece3.1683 (PMC6102530; doi:10.1002/ece3.1683)
Supplement: Supplementary file 2 — Appendix S1. List of passerine species. [file ECE3-5-5162-s002.docx]

| **SUPPLEMENTAL MATERIALS** | |  | |  | |
| --- | --- | --- | --- | --- | --- |
|  | |  | |  | |
| APPENDIX S1: List of passerine species | | | |  | |
|  | |  | |  | |
| Common Name | Scientific Name | | **Habitat Association** | |  |
| Acadian Flycatcher^1^ | Empidonax virescens | | mature deciduous, coniferous | |  |
| Bachman's Sparrow^2^ | Peucaea aestivalis | | mature pine and open habitat | |  |
| Black-throated Blue Warbler^1^ | Setophaga caerulescens | | undisturbed hardwood and mixed forest | |  |
| Blue-winged Warbler^1^ | Vermivora cyanoptera | | sapling, forest edge, clear cut | |  |
| Brewer's Sparrow^1^ | Spizella breweri | | sagebrush shrubland | |  |
| Brown-headed Nuthatch^2^ | Sitta pusilla | | pine forest | |  |
| Carolina Chickadee^2^ | Poecile carolinensis | | riverine habitat, swamp forest | |  |
| Cerulean Warbler^1^ | Setophaga cerulea | | forest | |  |
| Dickcissel^1^ | Spiza americana | | prairie grassland | |  |
| Eastern Towhee^3^ | Pipilo erythrophthalmus | | scrubby habitat, edge associated | |  |
| Eastern Wood-Pewee^1^ | Contopus virens | | wooded habitat | |  |
| Field Sparrow^1^ | Spizella pusilla | | brushy pasture, second groth scrub | |  |
| Fish Crow^2^ | Corvus ossifragus | | coastal, riverine | |  |
| Golden-winged Warbler^1^ | Vermivora chrysoptera | | partially open canopy and disturbance | |  |
| Gray Flycatcher^1^ | Empidonax wrightii | | sagebrush woodland, pine forest | |  |
| Green-tailed Towhee^1^ | Pipilo chlorurus | | shrubby habitat | |  |
| Henslow's Sparrow^3^ | Ammodramus henslowii | | grassland | |  |
| Hermit Warbler^1^ | Setophaga occidentalis | | coniferous | |  |
| Hooded Warbler^1^ | Setophaga citrina | | hardwood and edge habitat | |  |
| Indigo Bunting^1^ | Passerina cyanea | | shrubby areas, weedy fields | |  |
| Juniper Titmouse^2^ | Baeolophus ridgwayi | | juniper woodland | |  |
| Kentucky Warbler^1^ | Geothlypis formosa | | deciduous forest | |  |
| Louisiana Waterthrush^1^ | Parkesia motacilla | | streamside habitat with forest canopy | |  |
| Northern Parula^1^ | Setophaga americana | | canopy | |  |
| Pine Warbler^1^ | Setophaga pinus | | pine forest | |  |
| Prairie Warbler^1^ | Setophaga discolor | | fields, early successional | |  |
| Prothonotary Warbler^1^ | Protonotaria citrea | | water associated forest | |  |
| Sage Thrasher^1^ | Oreoscoptes montanus | | sagebrush shrubland | |  |
| Swainson's Warbler^1^ | Limnothlypis swainsonii | | forest | |  |
| Tufted Titmouse^2^ | Baeolophus bicolor | | deciduous forest | |  |
| Wood Thrush^1^ | Hylocichla mustelina | | forest | |  |
| Worm-eating Warbler^1^ | Helmitheros vermivorus | | forest | |  |
| Yellow-throated Vireo^1^ | Vireo flavifrons | | forest | |  |
| Yellow-throated Warbler^1^ | Setophaga dominica | | forest | |  |
|  | |  | |  |  |
| ^1^ neotropical migrant | |  | |  |  |
| ^2^ resident species  ^3^short distance migrant | |  | |  | |

**FIGURE LEGEND**

**FIGURE 1 SUPPLEMENTAL:**

Relationship between mean spring temperature at the realized niche margin (calculated as the 10 coldest/warmest routes with species occurrence during 1984-88) and mean spring temperature at the range margin expected to correspond to the niche edge (calculated as the 10 poleward / equatorward routes with species occurrence during 1984-88) at the a) cool edge and b) warm edge for neotropical migrants (n=26) and c) cool edge and d) warm edge for species bounded by the Gulf of Mexico at their equatorward margin (n=21). The black line represents an observed relationship, and the dashed line represents expected relationship if there is perfect correspondence between thermal niche limits and species’ range margin. Circular data points represent species that are shifting as expected based on temperature change. Temperature was corrected for total niche breadth.

**Figure 2 SUPPLEMENTAL:**

Temporal relationship in environmental distance calculated as the difference in mean spring temperature (°C) separating the thermal niche boundary and range margin in 1984-88 and 2002-06 for a) the poleward margin and cool niche boundary, and b) the equatorward margin and warm niche boundary for neotropical migrants (n=26), and c) the poleward margin and cool niche boundary, and d) the equatorward margin and warm niche boundary for species bounded by the Gulf of Mexico (n=21). The black line represents the observed relationship and the dashed line represents the expected relationship. Circular data points represent species that are shifting as expected given temperature changes. Temperature was corrected for total niche breadth.

**Figure 3 SUPPLEMENTAL:**

Probability of range margin shift based on local changes in mean spring temperature as a function of thermal niche proximity for a) poleward margin (log-likelihood = -21.36277, p=0.21), and b) equatorward margin (log-likelihood = -20.14979, p=0.059). Populations closer to warm niche limits were more likely to be lost through time, while colonization at the poleward margin was not related to the proximity of those populations to cold niche limits in the early time period. Expectations were based on the direction of climate change (warming or cooling) at occupied sites along range margins. Resident and short-distance migrants were excluded.

**Figure 4 SUPPLEMENTAL:**

Mean abundance change with change in temperature from 1984-1988 to 2002-2006 for a) cool thermal limit, b) warm thermal limit, and c) poleward, and d) equatorward margin. Sites occupied in 1984-1988 were held constant and absences in 2002-2006 were recorded as population extinction. Residents, short-distance migrants and neotropical migrants were included.

**Figure 5 SUPPLEMENTAL:**

Mean abundance change with change in temperature from 1984-1988 to 2002-2006 for a) cool thermal limit, b) warm thermal limit, c) poleward margin, and d) equatorward margin. Sites occupied in 1984-1988 were held constant and routes with absences in 2002-2006 were not included. Residents, short-distance migrants and neotropical migrants were included.
